# Borate‐Water‐Based 3D‐Slime Interface Quasi‐Solid Electrolytes for Li‐ion Batteries

**DOI:** 10.1002/adma.202505649

**Published:** 2025-07-09

**Authors:** Yosuke Shiratori, Kenta Watanabe, Kengo Saito, Ryota Sato, Yukihiro Okuno, Shintaro Yasui

**Affiliations:** ^1^ Analysis Technology Center FUJIFILM Corporation 210, Nakanuma Minamiashigara 250‐0193 Japan; ^2^ Present address: Laboratory for Zero‐Carbon Energy Institute of Science Tokyo 2‐12‐1, Ookayama Meguro‐ku Tokyo 152‐8550 Japan; ^3^ Laboratory for Zero‐Carbon Energy Institute of Science Tokyo 2‐12‐1, Ookayama Meguro‐ku Tokyo 152‐8550 Japan; ^4^ Materials and Structures Laboratory Institute of Science Tokyo 4259, Nagatsuta‐cho, Midori‐ku Yokohama 226‐8503 Japan

**Keywords:** aqueous lithium‐ion batteries, borates, direct recycling, hazardous materials‐free, non‐flammable, quasi‐solid‐state electrolytes, slime

## Abstract

The development of solid‐state batteries (SSBs) that do not use hazardous materials as electrolytes and are not flammable is progressing rapidly, however the production of sulfide‐based SSBs requires strict low‐dew‐point control due to their high reactivity with atmospheric moisture and the concern of generating hydrogen sulfide, and several issues remain in terms of the cost and recyclability. Thus, low‐cost facile materials and low‐CO_2_‐emission processes are necessary. With regard to oxide‐type SSBs, which are attracting attention for their safety, there are issues with manufacturing suitability, as high‐temperature sintering of oxide solid electrolyte particles is required. A new quasi‐solid‐state (QSS) electrolyte with 3D‐ionic conduction and adhesive interfaces by combining amorphous Li_2_B_4_O_7_ and water (3D‐Slime Interface Solid Electrolyte: 3D‐SLISE) is synthesized without stringent dew point control and sintering. Electrode and electrolyte slurries containing 3D‐SLISE are applied to current‐collecting foils in air, naturally dried, and used to construct battery laminates. 3D‐SLISE‐QSSBs (LiCoO_2_ cathode/3D‐SLISE with 7 wt.% bound‐water/Li_4_Ti_5_O_12_ or TiNb_2_O_7_ anodes) maintain several hundred cycles of charge/discharge as a 2.35 V lithium‐ion battery. The 3D‐SLISE‐QSSB technology can promote the use of safe and low‐cost batteries, eliminate the need for a dry room during manufacturing, and enable direct recycling of active materials.

## Graphical abstract

This advanced material, composed of lithium borate, lithium salt, and water, is a quasi‐solid that can be handled in air and has a 3D‐conducting‐interface network (3D‐Slime‐Interface Quasi‐Solid Electrolytes: 3D‐SLISE). The 3D‐SLISE is nonflammable and can be used in lithium‐ion batteries, and its water solubility allows direct recycling of active materials. This technology can contribute to the creation of a battery‐recycling society.

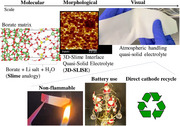

## 1 Introduction

Although the amount of primary energy consumed has been decreasing as electronics become more energy efficient, recently, the implementation of generation AI in society has led to an absolute increase in energy consumption. Then, batteries are extremely important for the efficient use of energy. The liquid‐type lithium‐ion batteries (LIBs) that are currently in widespread use require a manufacturing environment with a dew point of −40 °C or lower and flammable electrolytes with organic solvents.^[^ ^1^, ^2^, ^3^, ^4^ ^]^ In addition to safety issues, the construction of a battery recycling society is becoming an issue due to geopolitical risks.^[^ ^5^ ^]^

Solid‐state batteries (SSBs) are being actively studied and developed to improve performance and safety. SSBs comprising nonflammable sulfide‐based electrolytes, which exhibit excellent conductivity, could revolutionize the electric vehicle industry owing to their high energy density.^[^ ^6^ ^]^ However, the hydrolysis of sulfides by aerial moisture generates hydrogen sulfide, necessitating the utilization of special facilities with a dew point of −60 °C (or lower) for manufacturing SSBs.^[^ ^1^, ^2^ ^]^ The drying process for applied electrodes and the operation of dry rooms are highly energy‐intensive,^[^ ^2^ ^]^ and lowering these costs will directly reduce manufacturing expenses.^[^ ^7^ ^]^ Therefore, the development of moisture‐resistant electrolytes that can be used without rigorous dew point control could facilitate the fabrication of inexpensive and safe batteries.

The group of materials that can be synthesized by handling in air conditions are generally oxides. It is well known that the choices of oxide‐based electrolytes having higher ionic conductivity are perovskite‐type Li_x_La_y_TiO_3_ and garnet‐type Li_7_La_3_Zr_2_O_12_ families.^[^ ^8^ ^]^ However, the resistance at grain boundaries increases due to the hardness of oxide materials in comparison with sulfur‐based ones. To reduce this resistance, a high‐temperature sintering process must be needed. In this regard, this processing increases manufacturing costs and does not solve the fundamental problem. Therefore, as a new guideline, we focused on the synthesis of new electrolytes in complex materials.

Several studies on the concentrated organic and aqueous electrolytes have confirmed that the bonding of solvent molecules with anions effectively suppresses electrolysis,^[^ ^9^, ^10^, ^11^, ^12^, ^13^, ^14^, ^15^ ^]^ contributing significantly toward the development of safe aqueous batteries. These aqueous batteries retain at least 80% discharge capacity for several hundred cycles, contingent on the cathode–anode capacity ratio and charge/discharge rate.^[^ ^11^, ^13^, ^15^ ^]^ However, these are liquid‐based technologies, and when considering battery manufacturing, the electrolyte needs to be injected through a separator, which proves a barrier to further cost reduction and higher energy density. Electrolytes with excellent chemical stability and stacking suitability that can improve the energy density of SSBs have been extensively investigated. Quasi‐solid‐state (QSS) electrolytes for QSS batteries (QSSB) based on composites of ionic liquids (ILs) and oxide support‐particles or metal–organic frameworks have been reported to have a wide potential window,^[^ ^16^, ^17^, ^18^, ^19^, ^20^, ^21^, ^22^ ^]^ and aqueous QSS electrolytes.^[^ ^23^ ^]^ The characteristics of aqueous concentrated liquid electrolyte systems and quasi‐solid‐state battery systems are summarized in Table S1 (Supporting Information). Although previous studies have demonstrated the potential of aqueous battery systems, no technology has been reported that allows the so‐called all‐solid‐state battery process, in which all layers are applied and no electrolyte injection is required, to be performed in the general atmosphere. Furthermore, considering the direct recycling of the cathode active material, it is preferable to use a water‐soluble binder for the cathode.

Therefore, developing simple, cost‐effective (excellent recyclability), and chemically stable electrolytes with minimal environmental impact is crucial. As mentioned above, it is extremely difficult to create a solid electrolyte that satisfies all of the requirements of formability, ion conductivity, and atmospheric handling using a single material. To overcome these issues, we prepared a water‐based solid‐state lithium‐ion conductor (3D‐Slime Interface Quasi‐Solid Electrolytes: 3D‐SLISE), a composite material using lithium tetraborate (Li_2_B_4_O_7_) as a matrix, with reference to the chemistry of “Slime” made from borax (Na_2_B_4_O_7_⋅H_2_O).^[^ ^24^ ^]^ The lithium borate system originally comprises simple and light elements and is softer than most oxides used as matrix materials.^[^ ^25^, ^26^, ^27^ ^]^ Trilithium borate (Li_3_BO_3_) is used like a binder as a base material for Li‐ion conductors and exhibits Li‐ion conductivities in the order of 10^−5^ S cm^−1^ at 25 °C and 10^−4^ S cm^−1^ at 300 °C in Li_3_BO_3_–Li_2_SO_4_ glass ceramics and sintered Li_3_PO_4_–Li_3_BO_3_–Li_2_SO_4_ ternary ceramics, respectively.^[^ ^26^, ^27^ ^]^ However, high‐temperature sintering (at several hundred degrees Celsius) is required for fabricating lithium‐borate‐based ceramic solid electrolytes (which exhibit an ionic conductivity one to three orders of magnitude lower than that of other QSS electrolytes).^[^ ^16^, ^18^, ^19^, ^20^, ^21^, ^22^, ^23^ ^]^ Li_2_B_4_O_7_ is known as an optical‐crystal material,^[^ ^28^ ^]^ and was used as a Li‐conductive coating material to reduce the activation barrier between the electrolyte and active material.^[^ ^29^ ^]^ The softness of Li_2_B_4_O_7_ (a representative component of the lithium borate system) and the presence of BO_3_–BO_4_ chains (called the diborate structure) were the basis for its selection as a matrix material.^[^ ^30^ ^]^ None of them had the inspiration to apply the slime phenomenon characteristic of borax to electrolytes.

We explored chemically stable oxide‐based materials and atmospheric aqueous processes without stringent dew point control to develop cost‐effective and safe solid electrolytes for 2.4 V class QSSBs. Newly designed electrolytes (3D‐SLISE) aim to realize QSSBs suitable for auxiliary power sources in mobility, stationary storage batteries, and small consumer batteries.

## 2 Results and Discussion

### 2.1 A New Concept in Solid‐State Electrolytes

#### 2.1.1 Matrix Material

**Figure 1:**
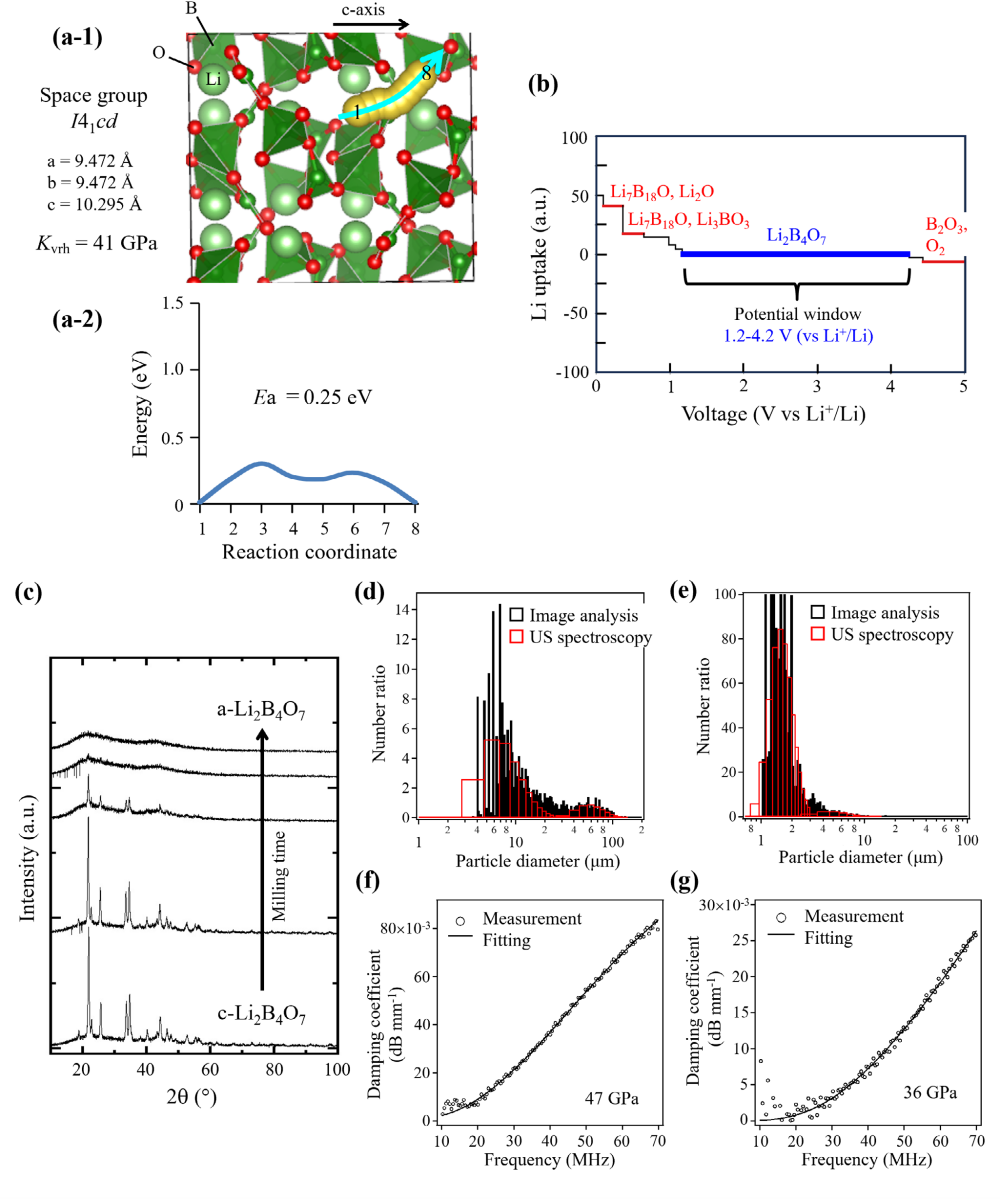
a‐1) Crystal‐structure model diagram of Li_2_B_4_O_7_ showing the lattice parameters, Voigt–Reuss–Hill elastic modulus (*K*_VRH_), and a‐2) active barrier for Li migration from coordinates 1 to 8 evaluated by ab‐initio calculations. b) Potential window for Li_2_B_4_O_7_ evaluated by ab‐initio calculations. c) XRD patterns indicating the amorphization of Li_2_B_4_O_7_ powder by mechanochemical treatment. Particle‐size distributions of d) crystal Li_2_B_4_O_7_ (c‐Li_2_B_4_O_7_) particles and e) amorphous Li_2_B_4_O_7_ (a‐Li_2_B_4_O_7_) particles, as indicated by image analysis and ultrasonic attenuation spectroscopy. Bulk modulus values of f) c‐Li_2_B_4_O_7_ particles and g) a‐Li_2_B_4_O_7_ particles were evaluated by the fitting of ultrasonic attenuation spectral data.

We prepared fine‐grained, low‐elasticity Li_2_B_4_O_7_ matrix materials with process suitability through mechanochemical processing. According to theoretical calculations, Li_2_B_4_O_7_ exhibits a low elastic modulus (41 GPa), low activation energy (0.25 eV) for Li‐ion conduction in the c‐axis direction (**Figure** **1**a), and high electrochemical stability within the potential window of 1.2–4.2 V (Figure 1b). The BO_3_‐BO_4_ chain (diborate structure like a polymer chain) is expected to form an ionic conduction layer and to function as a buffer phase against the expansion and contraction of the battery during operation. To soften crystal Li_2_B_4_O_7_ (c‐Li_2_B_4_O_7_), the crystals were amorphized via mechanochemical milling (Figure 1c). The c‐Li_2_B_4_O_7_ particles exhibit a wide particle‐size distribution of a few µm to 100 µm (Figure 1d), with a monotonically increasing ultrasonic attenuation spectrum in the range of 10–70 MHz (Figure 1f). In accordance with the scattering‐attenuation theory, the measured ultrasonic attenuation spectra are well fitted to the particle‐size distribution approximated by the Schultz distribution with a mean particle size of 8 and 63 µm, using the particle density and Poisson's ratio as input values. The bulk modulus and Young's modulus estimated from the fitting are 47 and 110 GPa, respectively. Figure 1e indicates that the amorphous‐Li_2_B_4_O_7_ (a‐Li_2_B_4_O_7_) particles show a narrower particle‐size distribution (of a few µm to 10 µm) than the c‐Li_2_B_4_O_7_ particles, with a monotonically increasing ultrasonic attenuation spectrum within 10–70 MHz (Figure 1g). The particle‐size distribution is approximated by the above theory with a mean size of 1.6 and 5.8 µm. The bulk modulus and Young's modulus evaluated from the fitting are 36 and 82 GPa, respectively. By amorphizing Li_2_B_4_O_7_ through mechanochemical processing, it was possible to reduce the bulk modulus from 47 to 36 GPa, while also narrowing the particle size distribution. a‐Li_2_B_4_O_7_ powder can be cold sintered (i.e., pressed into tough pellets under a uniaxial pressure of 200 MPa at 25 °C) owing to the presence of numerous active sites on the surface (which contains dangling bonded B atoms generated by the milling process).^[^ ^31^ ^]^ However, the ionic conductivity is on the order of 10^−9^ S cm^−1^, making it unsuitable for use in batteries.

**Figure 2:**
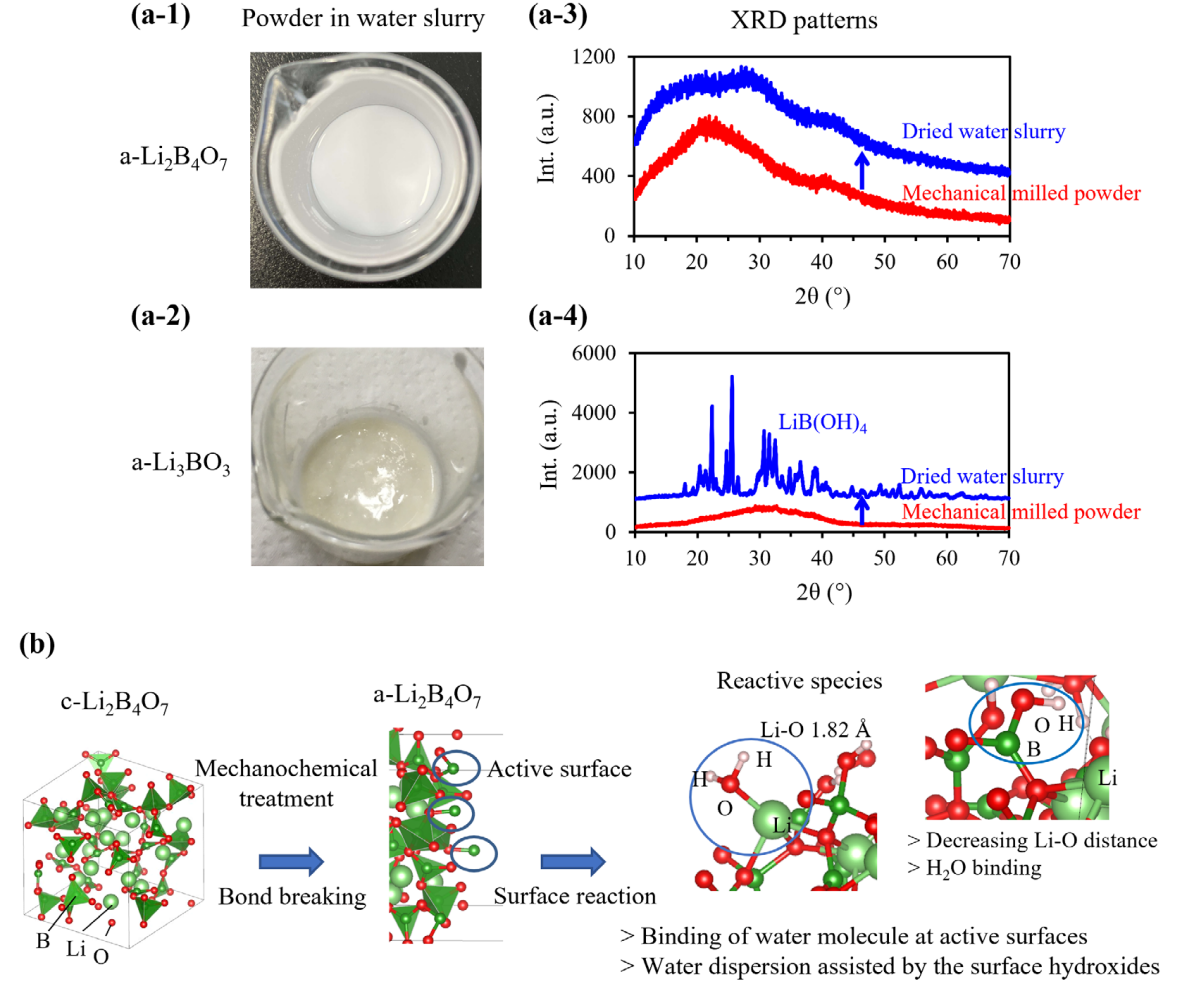
Water dispersibility of a‐1) a‐Li_2_B_4_O_7_ and a‐2) a‐Li_3_BO_3_ powders (47 wt.%), and XRD patterns of a‐3) a‐Li_2_B_4_O_7_ and a‐4) a‐Li_3_BO_3_ dried powders before and after water dispersion. b) Reaction model of the active surface of a‐Li_2_B_4_O_7_ derived by DFT‐MD calculations (8Li_2_B_4_O_7_ + 35H_2_O).

The mechanochemical treatment induces surface defects, rendering the particles metastable. In addition to H_2_O, a‐Li_2_B_4_O_7_ formed a homogeneous well‐dispersed slurry, whereas Li_3_BO_3_ (a‐Li_3_BO_3_) reacted strongly with H_2_O and crystallizes as LiB(OH)_4_ (**Figure** **2**a). Therefore, the latter cannot be used as an application slurry. Dangling bonds generated on the a‐Li_2_B_4_O_7_ surface by mechanochemical treatment function as H_2_O binding sites and dispersing groups. Notably, amorphous a‐Li_3_BO_3_ powder, the lithium borate that exhibits high potential as an additive for oxide‐type Li conductors,^[^ ^25^, ^26^, ^27^ ^]^ could not be dispersed in H_2_O. Li_3_BO_3_ comprises a triangular BO_3_^3−^ unit surrounded by Li‐ions, BO_3_ molecules exist in isolation (without binding to other BO_3_^3−^ molecules), and the *p* orbital of the B atom is empty, making it highly reactive. Therefore, the addition of Li_3_BO_3_ to H_2_O induces hydrolysis throughout the bulk structure to form LiB(OH)_4_. Because the structure of Li_2_B_4_O_7_ comprises a 3D network of triangular BO_3_ and tetrahedral BO_4_ (Figure 2b), the reaction of a‐Li_2_B_4_O_7_ with H_2_O is a surface phenomenon. Thus, a‐Li_2_B_4_O_7_ reacts with H_2_O mildly and disperses partially in a slime‐like state (without forming hydrate crystals).^[^ ^24^ ^]^ As shown in Table S2‐1 (Supporting Information), a‐Li_2_B_4_O_7_ reacts with water to form a gel that can be easily pelletized after drying at a low uniaxial pressure of 20 MPa. Gelation can be attributed to the polyborate reaction of borate ions in water.^[^ ^32^ ^]^ Dispersions of a‐Li_2_B_4_O_7_ powder with a gelatinized surface give solids with excellent binding properties after drying.

#### 2.1.2 3D‐SLISE

To utilize the unique properties of a‐Li_2_B_4_O_7_, slurries comprising a‐Li_2_B_4_O_7_, lithium bis(fluorosulfonyl)imide (Li(FSO_2_)_2_N; LiFSI) (to impart high Li‐ion conductivity),^[^ ^33^ ^]^ and carboxymethylcellulose (CMC; to improve the dispersion, viscosity, and adhesion of the slurry) were prepared (**Table** **1**). Slurries for electrodes (Type E) and for quasi‐solid‐electrolyte (QSE) layers (Type S) have different amounts of water to optimize the suitability of each slurry for application (Table S2‐5, Supporting Information). The dispersion stability of the slurry is slightly better in Type E than in Type S (Figures S1 and S2, Supporting Information). The viscosity of Type S is higher than that of Type E (Figure S2a,c, Supporting Information), making it optimal for QSE layer application without dripping. The low‐viscosity Type E is optimal as an electrolyte slurry for mixing with electrode compounds. In this way, each slurry is selected based on its physical properties for specific applications.

**Table 1:** Slurry compositions.

Slurries		Solid content [wt.%]	Slurry composition^a)^ [wt.%]/Molar ratio			
Type	Use		a‐Li_2_B_4_O_7_	LiFSI	CMC	H_2_O
E	for Electrode (anode and cathode)	79.9	37.3	41.1	1.5	20.1
			1.0	1.0	−	5.1
S	for quasi‐Solid‐electrolyte layer	69.8	32.6	35.9	1.3	30.2
			1.0	1.0	−	8.7

^a)^ The quasi‐solid‐state electrolyte “3D‐SLISE” introduced later contains ≈7 wt.% water as the ideal composition after removing excess water. The approximate molar ratio (a‐Li_2_B_4_O_7_/LiFSI/H_2_O) is as follows: 1.0/1.0/1.5.

The dynamic viscoelasticity of these slurries (Figure S2, Supporting Information) is similar to that of borax‐PVA hydrogels, i.e., “slime.”^[^ ^34^ ^]^ Type S and Type E were estimated to have a gel structure at the a‐Li_2_B_4_O_7_ matrix interface, namely an interface “slime” structure, formed by the interweaving of borate ions, water, and hydroxyl groups of CMC on the a‐Li_2_B_4_O_7_ surface of the matrix phase (Table S2, Supporting Information). In addition to the polybolate reaction between the a‐Li_2_B_4_O_7_ matrix surface and water described in 2.1.1, adsorption/cross‐linking of CMC on the matrix surface causes dispersion and structuring of the matrix particles in the slurry. Experimental evidence of these phenomena is shown in Figures S2–S4 (Supporting Information), and the structure of the slurry and dried solids is shown in Figure S5 (Supporting Information).

The reaction between the a‐Li_2_B_4_O_7_ surface, water molecules, and FSI anions and the formation of the interface structure were tracked by DFT‐MD calculations (Figure S6, Supporting Information). The a‐Li_2_B_4_O_7_ surface reacts immediately with water via the mechanism shown in Figure 2b, and Li coordinates with the oxygen atoms of water molecules, the a‐Li_2_B_4_O_7_ surface, and FSI anions. Details of the structure are shown in Figure S6 (Supporting Information). Furthermore, in addition to the strong Coulomb interaction between Li and the oxygen atoms of the FSI anion molecules, the FSI anion molecules were found to form hydrogen bonds with hydroxyl groups on a‐Li_2_B_4_O_7_ surface and H_2_O molecules (Figure S6, Supporting Information). This structure is similar to the interface model proposed for silica gel solid electrolytes (nanocomposites of silica and ionic liquids).^[^ ^19^ ^]^ However, there are some differences, such as the presence of Li atoms on the surface of a‐Li_2_B_4_O_7_, which strongly anchor H_2_O and LiFSI molecules through Coulomb interactions.

**Figure 3:**
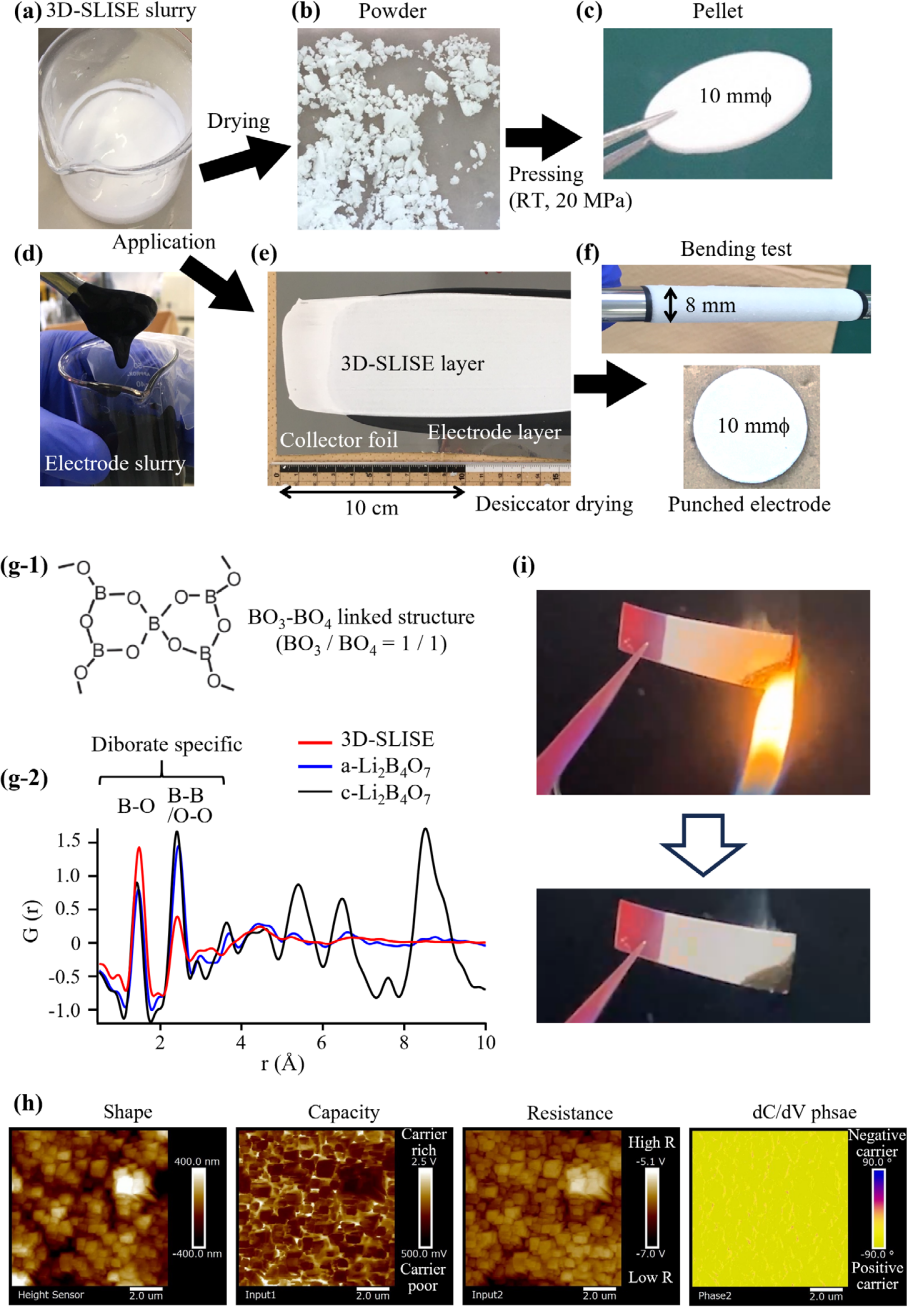
Various aspects of the different forms of 3D‐SLISE. a) 3D‐SLISE slurry, b) 3D‐SLISE dry powder, c) a 3D‐SLISE pressed pellet, d) electrode slurry containing 3D‐SLISE, e) a multilayer application film comprising 3D‐SLISE on a cathode, f) bending test of the multilayer application film, and an electrode punched from the multilayer application film (10 mmϕ, containing 15.0 mg of LiCoO_2_). g‐1) Schematic image of diborate BO_3_‐BO_4_ linked structure. g‐2) Reduced two‐body distribution functions of 3D‐SLISE, amorphous Li_2_B_4_O_7_ (a‐Li_2_B_4_O_7_), and crystalline Li_2_B_4_O_7_ (c‐Li_2_B_4_O_7_) constructed using X‐ray total‐scattering data. h) Scanning microwave impedance microscopy image (shape, capacitive component, resistive component, and dC/dV phase) of the 3D‐SLISE surface (under nitrogen). i) Flame test for 3D‐SLISE (under air at 25 °C, 30% RH).

Excess water was removed from the prepared slurry and evaluated in various forms (**Figure** **3**). These composites (comprising a lithium borate matrix phase, water, and Li salt) were labeled 3D‐Slime Interfacial Quasi‐Solid Electrolyte (3D‐SLISE). This designation is derived from the fact that the soft interfacial carrier‐rich layers spread three‐dimensionally in a mesh‐like pattern inside the solid to form conduction pathways, as shown below. The 3D‐SLISE slurry is neutral (pH ≈7) and does not contain any visible agglomerates (Figure 3a), and a thin layer (achieved thickness: 9 ± 1 µm) of 3D‐SLISE dries and solidifies spontaneously in air at a temperature of 25 °C and relative humidity (RH) of 30% or lower (Figure S7a, Supporting Information). Moreover, the 3D‐SLISE slurry exhibits no property changes after four months of refrigerated storage (Figure S7b, Supporting Information). The good water dispersibility of a‐Li_2_B_4_O_7_ particles can be attributed to their abundant surface hydroxyl groups, as shown in Figure 2b.

Dried 3D‐SLISE powder (Figure 3b) was pelletized under a uniaxial pressure of 20 MPa (Figure 3c). Figure 3d,e shows the electrode slurry and the multilayer film (uniform, with no agglomerates) fabricated by the application and spontaneous/natural drying of the 3D‐SLISE slurry on the electrode film. The applied electrode does not exhibit cracking or edge‐chipping during bending and punching tests (Figure 3f). 3D‐SLISE, with a B‐based composition of Li_2.9_B_4_O_18.2_F_2.0_S_2.4_Na_0.1_, is a solid that contains a significant amount (several wt.%) of bound water. The Na in 3D‐SLISE is derived from the CMC additive used during its synthesis. 3D‐SLISE is amorphous owing to the loss of long‐range order; however, the presence of B–O and B–B short‐range order in 3D‐SLISE preserves the diborate structure (Figure 3g). LiFSI exists in an amorphous state (Figure S8, Supporting Information) and, therefore exhibits ionic conductivity. Scanning microwave impedance microscopy images of the 3D‐SLISE film surface (Figure 3h) reveal submicron particles surrounded by carrier‐rich layers. These layers, containing positive carriers (dC/dV phase), exhibit lower resistance than the interiors of the submicron particles. Moreover, these interface layers have solid elasticity (Figure S9, Supporting Information) and contain relatively large amounts of LiFSI and water, enabling them to function as excellent quasi‐solid interfaces that provide both adhesion and conductivity. These carrier‐rich interfaces spread three‐dimensionally in the solid to form conduction paths. The formation of 3D networks can be attributed to the reaction between Li_2_B_4_O_7_, H_2_O, and CMC molecules, and to the hydrogel properties of a‐Li_2_B_4_O_7_ [hydrolysis and self‐condensation like borax, Table S2‐1 (Supporting Information)].^[^ ^24^, ^35^ ^]^ The involvement of boron in the formation of the interface conduction interface in 3D‐SLISE (polyborate reaction, etc., Table S2‐1, Supporting Information) is supported by composition analysis targeting the interface (Figure S10, Supporting Information). During the flame test under air for 3D‐SLISE,^[^ ^23^ ^]^ the flame does not spread when it comes in contact with 3D‐SLISE (Figure 3i). Therefore, safe battery operation is possible when 3D‐SLISE is used as an electrolyte.

### 2.2 Electrochemical Properties of 3D‐SLISE

**Figure 4:**
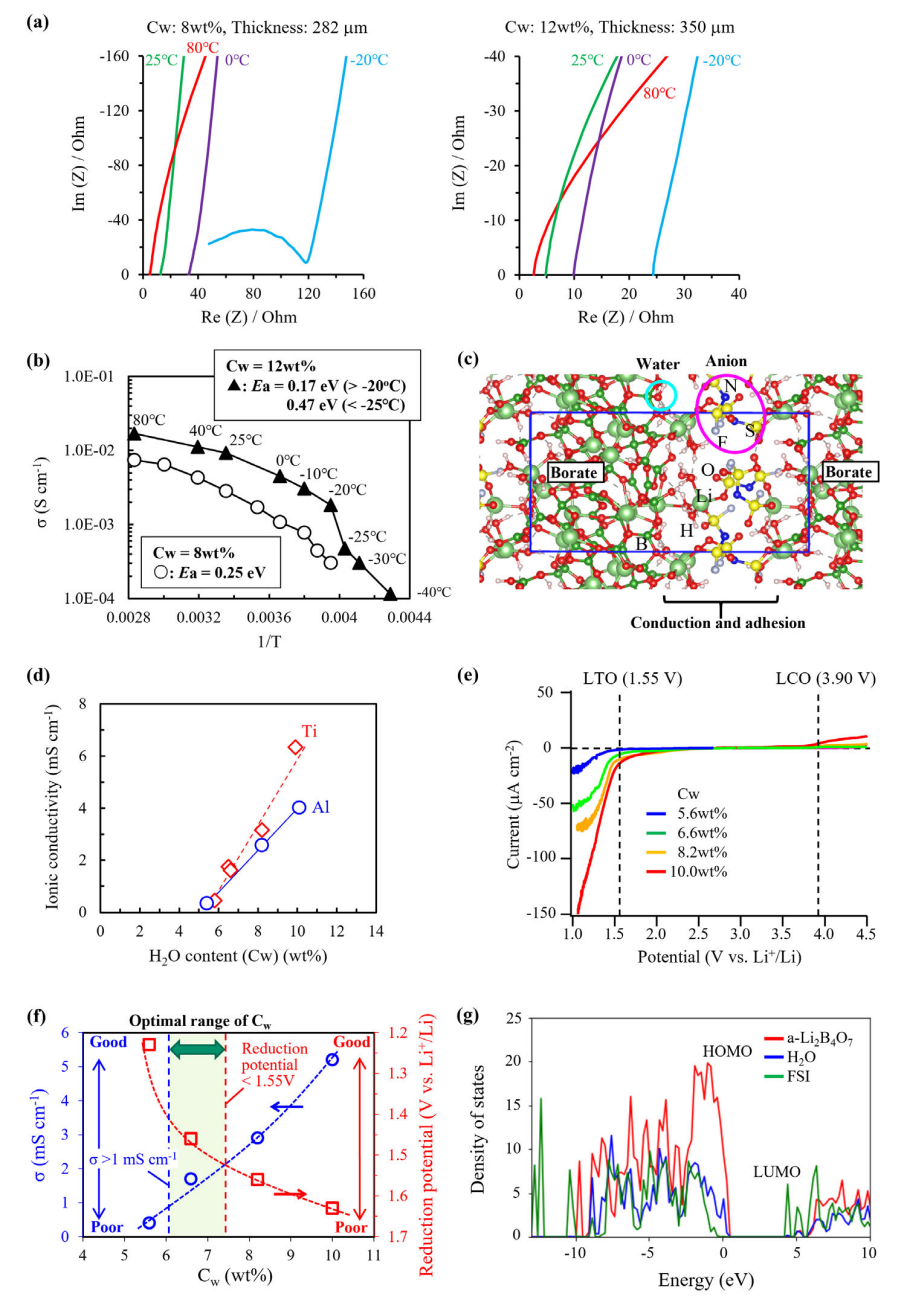
a) Impedance spectra of 3D‐SLISE with the water content values (C_w_) of 8 and 12 wt.%. b) Temperature dependence of ionic conductivity. c) A snapshot of DFT‐MD calculations for the 3D‐SLISE structural model. d) Relationship between C_w_ and ionic conductivity of 3D‐SLISE (with Ti foil sandwich and Al metal foil sandwich). e) Linear sweep voltammograms of 3D‐SLISE with different C_w_ values. f) Relationship between the water content (C_w_), ionic conductivity (σ), and reduction potential (V vs Li^+^/Li) of 3D‐SLISE. (g) Partial density states (PDOS) of H_2_O, FSI, and a‐Li_2_B_4_O₇ derived from DFT‐MD calculations for the 3D‐SLISE structural model.

**Figure** **4**a,b illustrates the temperature dependence of the electrochemical impedance spectra and ionic conductivity of 3D‐SLISE with water contents (C_w_) of 8 and 12 wt.%. Both samples exhibit a conductivity transition at low temperatures, particularly at −22 °C, with the 8 wt.% sample showing a significant drop in conductivity. This transition may be attributed to the crystallization of isolated LiFSI, which is inhibited at high water contents. The activity barriers (*E*_a_) above −20 °C are 0.25 and 0.17 eV for 3D‐SLISE, with C_w_ values of 8 and 12 wt.%, respectively. These values are comparable to Water‐in‐Salt electrolytes (≈0.23 eV in 21 M LiTFSI‐H_2_O) and significantly smaller than the literature value of *E*_a_ for oxide‐type solid‐state electrolytes (≈0.4 eV).^[^ ^36^, ^37^ ^]^ This can be attributed to the excellent adhesion and conduction interface comprising a‐Li_2_B_4_O_7_, LiFSI, and H_2_O (Figure 4c; Figure S6, Supporting Information). 3D‐SLISE with a C_w_ of 8 wt.% exhibits a slightly larger *E*_a_ than the sample with a C_w_ of 12 wt.% owing to the former higher hardness. The relationship between C_w_ and the conductivity of 3D‐SLISE is shown in Figure 4d. The slope varies with the metal foil used and the ionic conductivity increases with the water content. The tendency of the conductivity to decrease on using an Al foil can be partially attributed to a slight increase in resistance caused in part by the reaction between 3D‐SLISE and Al surface despite the pH of the 3D‐SLISE slurry is neutral.

Linear sweep voltammograms of 3D‐SLISE with different C_w_ and the redox potentials of the cathode (LiCoO_2_; LCO) and anode (Li_4_Ti_5_O_12_; LTO) active materials (3.90 and 1.55 V, respectively) are shown in Figure 4e. The redox potential (edge of the potential window) was defined as the potential (in the voltammogram) at which an oxidation or reduction current of 10 µA cm^−2^ was observed. The C_w_, ionic conductivity, and potential window are summarized in **Table** **2**. At any C_w_ value, the oxidation side was sufficiently stable for the Li^+^‐insertion/desorption reaction of LCO. On the reducing side, 3D‐SLISE decomposition significantly occurred at a C_w_ of 10 wt.% (because the potential was 0.08 V higher than that of the Li^+^‐insertion/desorption reaction of LTO). Figure 4f shows the optimal range of C_w_ that satisfies the conditions of an ionic conductivity of 1 mS cm^−1^ or more and a reduction potential of 1.55 V or less. It is preferable to adjust C_w_ to ≈7%.

**Table 2:** Water content, ionic conductivity, and potential window of 3D‐SLISE.

C_w_ [wt.%]		5.6	6.6	8.2	10.0
Ionic conductivity at 25 °C (mS cm^−1^)		0.4	1.7	2.9	5.2
Potential window (V)	Oxidation side	>4.50	>4.50	>4.50	4.45
	Reduction side	1.23	1.46	1.56	1.63

According to the density of electronic states of a‐Li_2_B_4_O_7_/(H_2_O + FSI) evaluated by DFT calculation (Figure 4g; details are shown in Figure S6g,h, Supporting Information), the highest occupied molecular orbital (HOMO) of a‐Li_2_B_4_O_7_ is located at a higher energy than the HOMO of H_2_O, indicating that oxidation of H_2_O does not occur preferentially. Hydration of Li‐ions (i.e., pinning of H_2_O) lowers the HOMO level of H_2_O, thereby increasing its oxidation potential.^[^ ^14^ ^]^ Furthermore, the lowest unoccupied molecular orbital (LUMO) of the FSI anion is energetically lower than that of H_2_O. This enables the reduction of FSI anions prior to that of H_2_O molecules. High‐concentration Li‐salt electrolytes typically exhibit the preferential reduction of anions.^[^ ^14^, ^15^ ^]^

The transport of Li‐ions is then discussed by modeling the interfacial conduction layer. Mean square displacement (MSD) of Li, H_2_O, and FSI molecules at 300 K for a) 28 b) 20 (c) 10 mol kg^−1^ LiFSI/H_2_O eutectic system as a model system of the conductive interface in 3D‐SLISE were calculated by using machine learning potential molecular dynamics (MLP‐MD) (Figure S11, Supporting Information).^[^ ^38^ ^]^ Note that the analysis of the interface (Figures S6 and S10, Supporting Information) suggests that the a‐Li_2_B_4_O_7_ surface is involved in the formation of the conduction interface, however for simplicity of the conduction interface model, a‐Li_2_B_4_O_7_ is not included.

The self‐diffusion coefficients of Li, H_2_O, and FSI were estimated from the slope of the MSD (Table S3, Supporting Information). As the H_2_O content increases, the diffusion of Li‐ions also increases, as indicated by the computational results. As the relative content of H_2_O increases, the influence of FSI molecules that hinder the movement of water molecules diminishes, leading to increased mobility of the water molecules. Consequently, the Li‐ions, which are strongly bound to the water molecules by Coulombic forces, are also thought to exhibit higher diffusivity due to the kinetic energy transfer from highly mobile water molecules. This picture is consistent to the observed Li‐ion diffusion coefficients from pulsed field gradient NMR. The diffusion coefficients in 3D‐SLISE (C_w_ = 7, 10wt.%) were estimated by pulsed field gradient ^7^Li‐NMR and ^19^F‐NMR spectroscopy (Figure S12, Supporting Information). The obtained coefficients are shown in **Table** **3**. The Li^+^ transport number [t_Li+_ = D_Li+_/(D_Li+_ + D_FSI‐_)] for both samples was estimated to be ≈0.6, with an error of less than 0.02. This value is approximately the same as that of a concentrated LiFSI electrolyte (20 mol kg^−1^ aq.). The pulsed field gradient NMR method evaluates the diffusion behavior of ions over a diffusion distance of ≈1 µm and a diffusion time of 100 ms. The analysis of the Li^+^ transport number suggests that there is a common Li^+^ conduction mechanism in 3D‐SLISE and LiFSI concentrated electrolytes over the above spatial and temporal scales.

**Table 3:** Ion diffusion coefficients and Li^+^ transport numbers in 3D‐SLISE were obtained using pulsed field gradient NMR (weight ratio: L_2_B_4_O_7_/LiFSI/H_2_O = 48/52/x).

	3D‐SLISE		LiFSI [20 mol kg^−1^ aq.]
C_w_ (wt.%)	7	10	−
D_Li_^+^ [m^2^ s^−1^] × 10^−11^	1.85 ± 0.07	3.03 ± 0.11	13
D_FSI_^−^ [m^2^ s^−1^] × 10^−11^	1.21 ± 0.05	2.30 ± 0.13	8.3
t_Li_^+^	0.60 ± 0.01	0.57 ± 0.02	0.61

The concentration of 28 mol kg^−1^ (Figure S11a, Supporting Information) corresponds roughly to the LiFSI/water composition ratio in 3D‐SLISE with an optimal amount of water (7 wt.%). At 28 mol kg^−1^, most of the water molecules were isolated without forming clusters (Figure S13a, Supporting Information). Li‐ions were found to bind and exchange with oxygen of water and oxygen of FSI anions (Figure S14, Supporting Information). These results suggest that water is approximately fixed in 3D‐SLISE with 7% water content and that the conduction mechanism of Li‐ions is close to the hopping‐type.

### 2.3 Integration of 3D‐SLISE into Batteries

**Figure 5:**
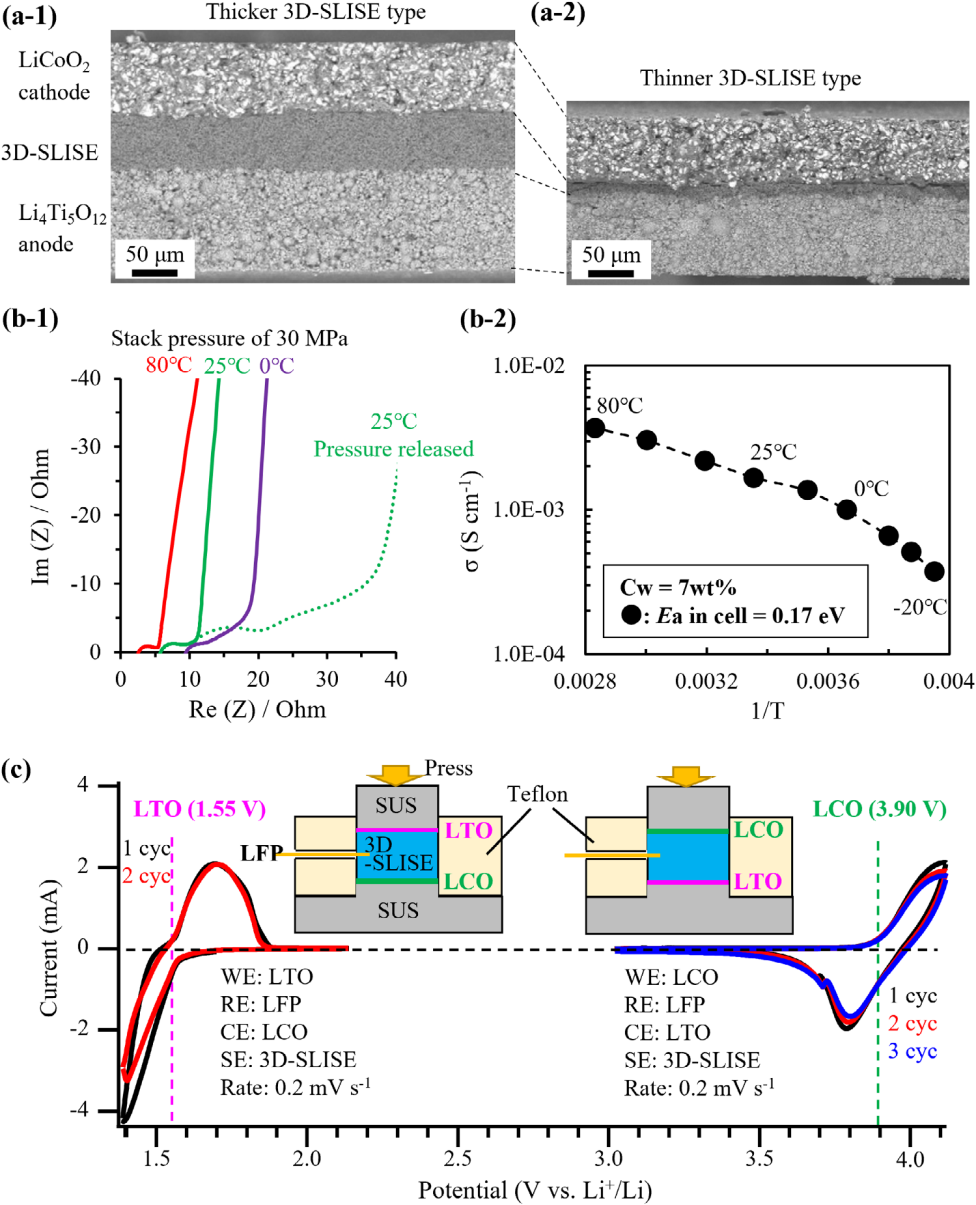
Cross‐sectional SEM images of 3D‐SLISE‐QSSBs with a‐1) thicker 3D‐SLISE layer and a‐2) thinner 3D‐SLISE layer. b‐1) Impedance spectra and b‐2) Arrhenius plot of ionic conductivity of the 3D‐SLISE layer in the battery estimated from bulk resistance (C_w_ of 3D‐SLISE layer = ≈7 wt.%). c) cyclic voltammograms (C‐V) of the cathode and anode at 27 °C under a stack pressure of 30 MPa. Schematic illustrations in the inset are the C‐V measurement system of three‐electrode cell.

**Figure** **5**a shows the cross‐sectional morphology of laminates comprising 3D‐SLISE applied layers with thicknesses of ≈60 and ≈20 µm (Figure 5a‐1,a‐2, respectively). The [cathode layer]/[3D‐SLISE layer]/[anode layer] is uniformly fabricated without short circuits. The temperature dependence of the electrochemical impedance spectra and ionic conductivity of uniaxial 30 MPa‐stacked 3D‐SLISE‐QSSBs (with a 60 µm 3D‐SLISE layer) are shown in Figure 5b‐1,b‐2. The 3D‐SLISE‐QSSBs exhibit a bulk resistance of 5.7 Ω at 25 °C (at the rising edge of the impedance spectrum) with an effective area of 0.5 cm^2^. The water content of 3D‐SLISE (C_w_) in the battery was estimated to be 7 wt.% using the method shown in the Experimental Section. 3D‐SLISE with a C_w_ of 7 wt.% exhibits an ionic conductivity of 2.1 mS cm^−1^ (estimated using the relationship shown in Figure 4d). Moreover, 3D‐SLISE layers in cells exhibit the same temperature dependence as isolated 3D‐SLISE samples. On releasing the uniaxial stack pressure, arcs on the low‐frequency side (with diameters of ≈10 and 20 Ω) become apparent. This is possibly a solid‐specific phenomenon originating from the resistance of the component interface. Cyclic voltammograms of the cathode and anode (C_w_ of the 3D‐SLISE layer is ≈7 wt.%) are shown in Figure 5c. Here, the amount of active material in the counter electrode (CE) is significantly larger than that in the working electrode (WE). Therefore, the current is limited by the electrochemical reaction in the WE. Reversible redox reactions of the cathode (LCO) and anode (LTO) active materials are detected at 3.90 V (vs Li^+^/Li) and 1.55 V (vs Li^+^/Li), respectively. On the anode side, a large reduction current is recorded in the first cycle, which decreases in the second cycle owing to the reduction decomposition of some 3D‐SLISE in the first cycle. These results and the potential window of 3D‐SLISE (Figures 4, 5, Table 1) confirm the high potential applicability of 3D‐SLISE in the 2.4 V‐class LCO/LTO batteries.

### 2.4 3D‐SLISE‐QSSB Properties

**Figure 6:**
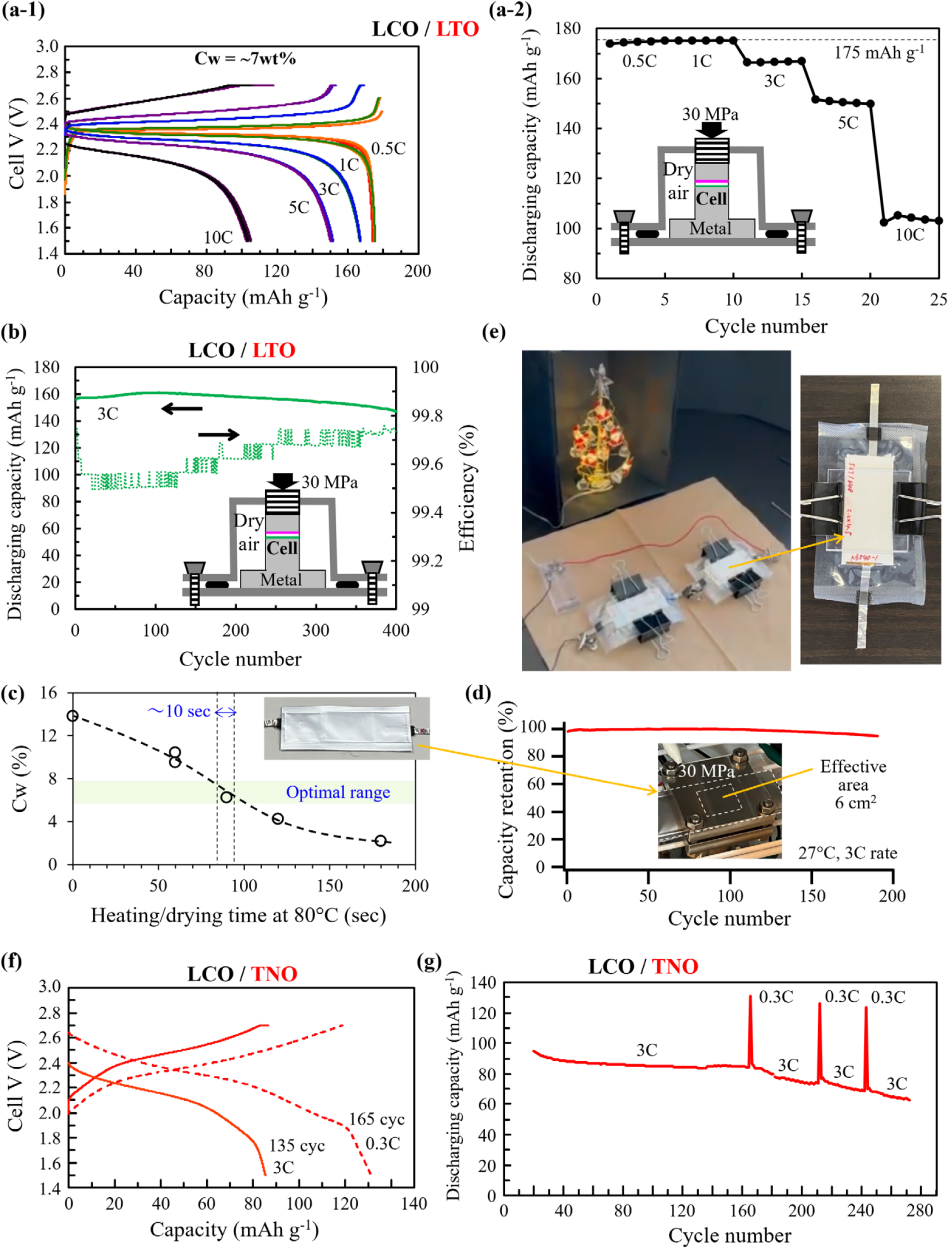
a‐1) Charge–discharge curves under several C‐rate of a 3D‐SLISE‐QSSB (C_w_ of the 3D‐SLISE layer = ≈7 wt.%, ratio of cathode and anode capacities: P/N = 1.7 (anode rate‐limiting), LCO: 16.8 mg cm^−2^, LTO: 7.7 mg cm^−2^) recorded under a uniaxial stack pressure of 30 MPa using a commercial SSB fixture at 27 °C. a‐2) Discharge capacity versus cycle number plotted using data of charge–discharge curves. Constant current (CC)–constant voltage (CV) charge and CC discharge (with a lower voltage limit of 1.5 V) with an open‐circuit time of 10 min are used for rate tests. The following upper voltage (for CC charge) and lower current (for CV charge) limits are used at 0.5C: 2.5 V/0.45C; 1C: 2.6 V/0.8C; 3C: 2.7 V/2.4C; 5C: 2.7 V/4C; and 10C: 2.7 V/8C. b) Cycling characteristics of the aforementioned battery at 3C and 27 °C. A schematic illustration of the measurement system is shown in insets of a‐2 and b. c) Relationship between heating/drying time at 80 °C during the fabrication of laminated cells (shown in inset) and the water content of 3D‐SLISE (C_w_). d) Cycling characteristic of the laminated cell (shown in inset) at 3C rate at room temperature (P/N = 3.3). e) Lighting test of a white LED using a skeleton‐type battery (in series) prepared using 3D‐SLISE as an electrolyte under 30% RH. Schematic of the fabrication procedure and evaluation results are shown in Figures S15 and S16 (Supporting Information), respectively. f) Room temperature rate characteristics of an 3D‐SLISE battery (C_w_ of the 3D‐SLISE layer = ≈7 wt.%, the ratio of anode and cathode capacities: N/P = 1.6 (cathode rate‐limiting), LCO: 14.1 mg cm^−2^, TNO: 8.4 mg cm^−2^) recorded under a uniaxial stack pressure of 30 MPa using a commercial SSB fixture at 27 °C. Constant current (CC)–constant voltage (CV) charge and CC discharge (with a lower voltage limit of 1.5 V) with an open‐circuit time of 10 min are used for rate tests. The following upper voltage (for CC charge) and lower current (for CV charge) limits are used at 0.3C: 2.7 V/0.24C and 3C: 2.7 V/2.4C. g) Room temperature cycling characteristics of the aforementioned battery at 3C and 27 °C.

The charge–discharge rate characteristics of a 3D‐SLISE‐QSSB (C_w_ of the 3D‐SLISE layer = ≈7 wt.%) evaluated using a commercially available QSSB fixture and a uniaxial stack pressure of 30 MPa are shown in **Figure** **6**a. Under the anode‐rate‐limiting condition, the theoretical capacity of LTO (175 mAh g^−1^) is discharged at 0.5C and 1C, and 57% of the theoretical capacity is discharged at 10C. Under the experimental conditions used in this study (single‐cell standard, including current collectors), the gravimetric energy density is estimated to be 50 Wh kg^−1^. There is room for improvement in the future by reducing the capacity ratio of cathode and anode (P/N), making the current collector thinner, and adopting a bipolar structure. The cycling characteristics at 3C and 27 °C are shown in Figure 6b. The capacity slightly increases up to 100 cycles and then gradually decreases, with an increase in the coulombic efficiency. A similar phenomenon is reported for gel‐based QSSBs and liquid‐based LIBs that utilize LTO as the anode active material.^[^ ^23^, ^39^ ^]^ The reductive decomposition of excess H_2_O molecules and FSI^−^ at the anode triggers the formation of a 3D‐SLISE/LTO interface and increases the active material utilization, thereby increasing the discharge capacity. As the interfacial layer further thickens, the side reactions are suppressed, and the coulombic efficiency increases, resistance increases, and discharge capacity decreases. The 3D‐SLISE‐QSSB retains 92% capacity after 400 cycles, comparable to batteries using established aqueous electrolytes.^[^ ^13^, ^15^, ^23^ ^]^ Water content of 3D‐SLISE (C_w_) in laminated batteries, which were designed for practical use, was also successfully controlled to the desired level (≈7 wt.%) through vacuum packaging immediately after the heating and drying process under atmospheric conditions shown in Figure 6c, and charge/discharge cycling test was demonstrated (Figure 6d). Moreover, skeleton‐type batteries (packaged in a transparent plastic film and pressed by a clip) were produced under atmospheric conditions, lighting a white LED when connected in series (Figure 6e). The prepared battery operates under low stack pressure owing to the flexibility and interfacial adhesion of 3D‐SLISE. The applicability of high‐capacity TiNb_2_O_7_ (TNO) as an anode active material was evaluated. TiNb_2_O_7_ has an electrode potential of 1.6 V versus Li⁺/Li, which is almost the same as LTO, and has a theoretical capacity of 387 mAh g^−1^ and 1680 mAh cm^−3^, which is extremely large compared to LTO (175 mAh g^−1^, 580 mAh cm^−3^).^[^ ^40^ ^]^ As in the case of LTO, it was possible to make aqueous slurry, apply it, and fabricate 3D‐SLISE‐QSSBs. The measured charge/discharge characteristics (cathode rate‐limiting) are shown in Figure 6f. The horizontal axis is the capacity per unit weight of the cathode active material (LCO). The charge/discharge curve reflects the characteristic potential behavior of TNO.^[^ ^40^ ^]^ Since degradation progresses more when low‐rate charging and discharging are performed (Figure 6g), it is thought that under these conditions, the decomposition reaction becomes more apparent than the battery reaction. Through structural optimization of peripheral components (e.g., reducing the weight of the current collector, adopting a bipolar structure), 3D‐SLISE‐QSSBs that use TNO can reach 100 Wh kg^−1^. Further increases in capacity are currently being investigated by optimizing the active material and N/P.

### 2.5 Degradation Mechanism

**Figure 7:**
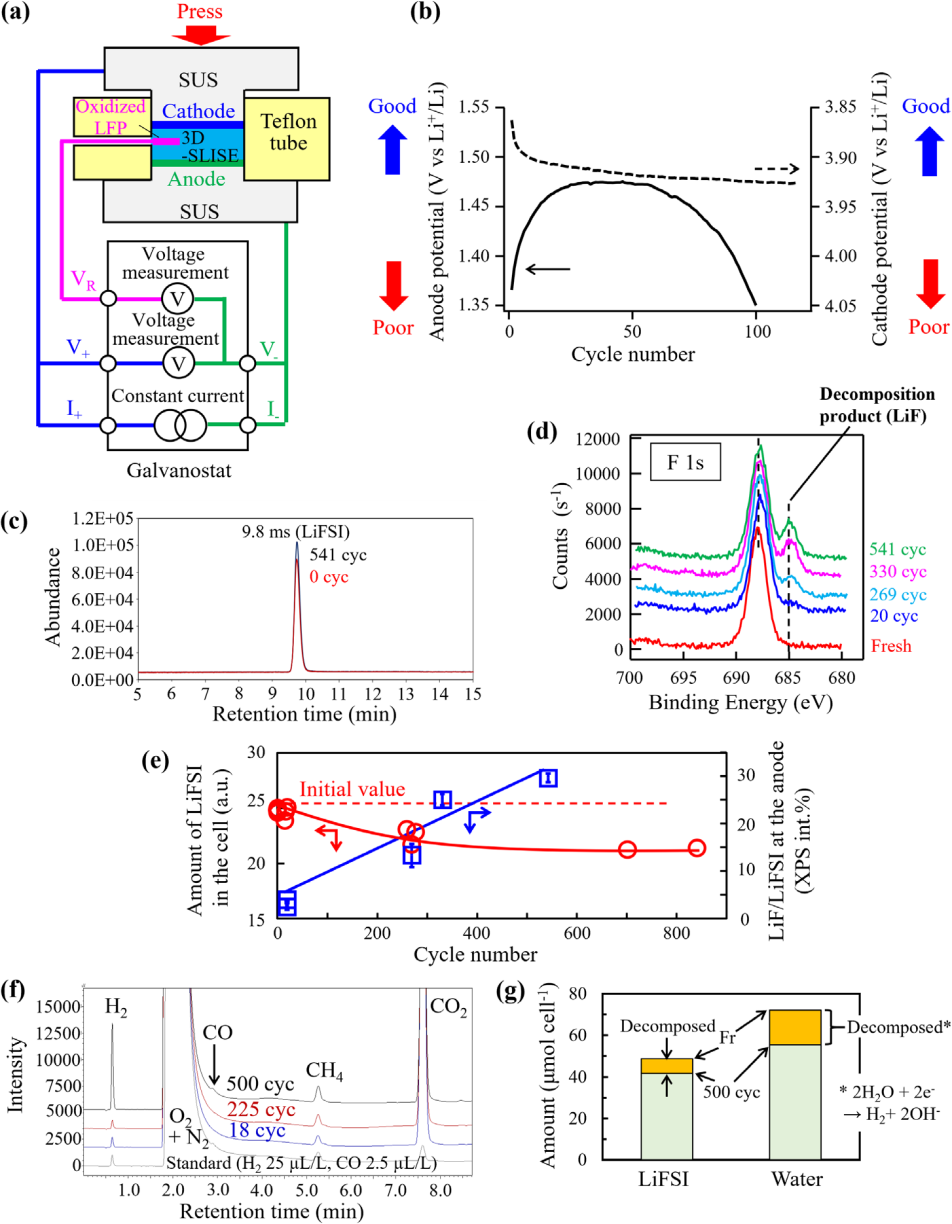
a) Schematic of a triode test. b) Cycle dependence of the anode and cathode potentials (charge potential at 50 mAh g^−1^) under accelerated degradation conditions with a C_w_ = 10 wt.% or more (27 °C, P/N = 1.4, 3C rate). c) Selected ion monitoring (SIM) chromatogram of the components extracted from the battery after the cycle test. d) F1s X‐ray photoelectron spectra (XPS) of fluorine obtained on the anode side of the interface between the anode and the Al current collector foil of batteries that decomposed under non‐atmospheric exposure conditions after cycle tests. e) The cycle dependence of the amount of LiFSI in the battery as determined by liquid chromatography‐mass spectrometry (LC‐MS) and the XPS intensities of LiF and LiFSI in the anode removed after the cycle test. The cycle test was conducted under the following conditions: 27 °C, P/N = 3.3, lower limit voltage: 1.8 V, upper limit voltage: 2.55 V, charge: CCCV, discharge: CC, rate: 3C. f) GC/BID chromatograms of gas collected from the battery test jigs after the cycle tests. g) The amount of LiFSI and water in the battery before and after the cycle test. The amount that decreased due to decomposition is shown in orange.

The anode degradation, identified through triode charge/discharge evaluation (**Figure** **7**a), is the primary cause of the battery degradation. The initial increase in discharge capacity (improvement) is due to a decrease in overvoltage at the LTO interface (increase in electrode reaction rate) (Figure 7b). The later decrease (deterioration) is due to an increase in overvoltage caused by the partial decomposition of 3D‐SLISE at the anode. During cycling, the amount of LiFSI decreases while that of LiF, a decomposition product, increases in the anode (Figure 7c–e). This phenomenon is not observed on the cathode. Gas collected from the battery test jigs after the cycle tests was analyzed (Figure 7f). The amount of gas generated per cycle was 0.68 µL cycle^−1^ for the 18 cycles sample and 0.37 µL cycle^−1^ for the 500 cycles sample, with the amount generated being higher at the beginning. CO was detected as a unique gas, however the amount generated was ≈1/100 compared to H_2_. If all hydrogen extracted after 500 cycles (quantified using GC/BID) is considered to originate from water reduction, 23% (or 16.7 µmol) of the initial water is found to decompose (Figure 7g). Thus, the degradation stems from the LiFSI and water decomposition at the anode. Efforts to mitigate decomposition by modifying the anode material are ongoing and will be reported separately.

### 2.6 Environmental Friendliness and Recyclability of the 3D‐SLISE Technology

**Figure 8:**
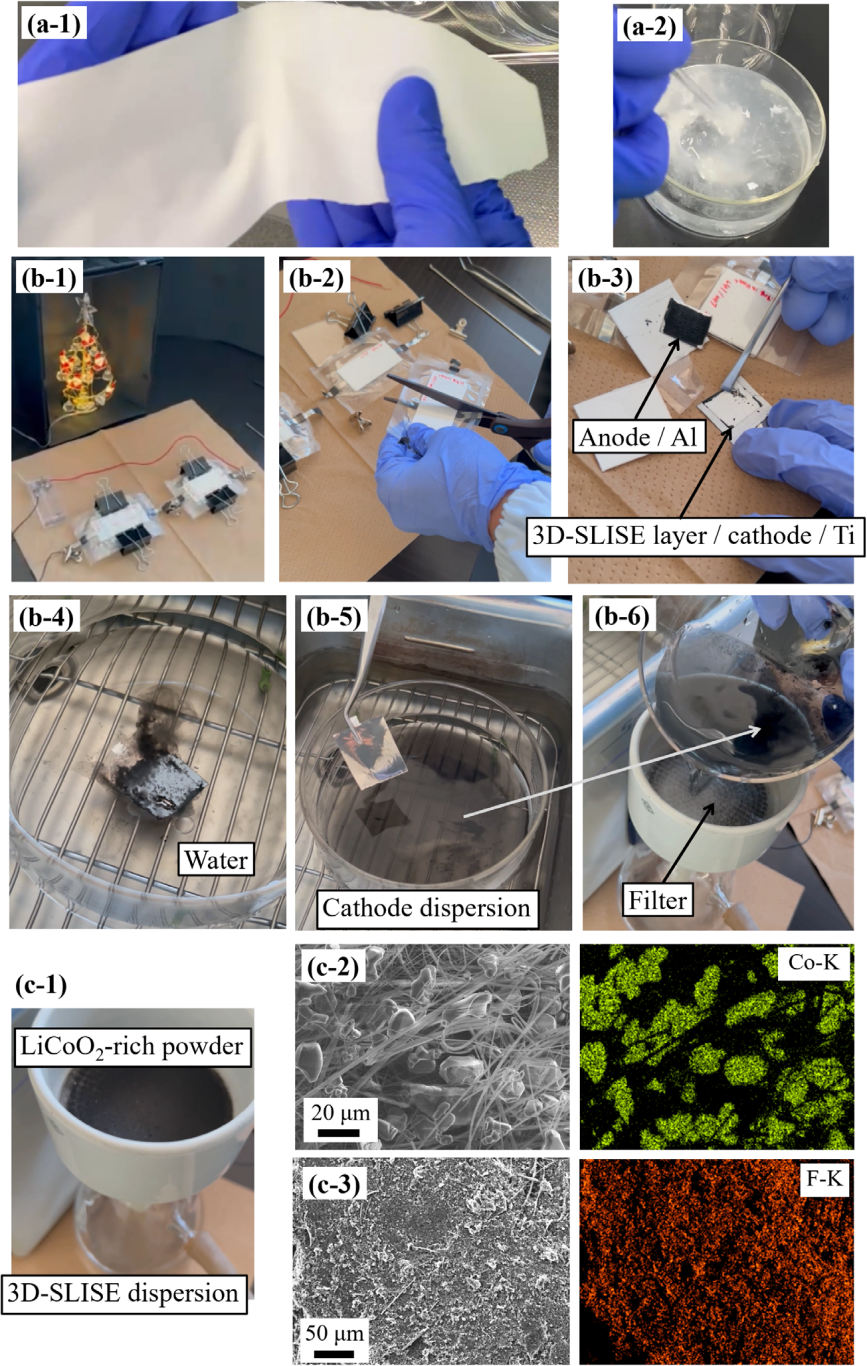
a‐1) 3D‐SLISE self‐supporting sheet; a‐2) dissolution of 3D‐SLISE self‐supporting sheet in water (Movie S1, Supporting Information); b‐1) lighting test of white LED using skeleton‐type battery (in series) made with 3D‐SLISE as electrolyte under 30% RH (Movie S2, Supporting Information); b‐2) cutting a skeleton battery using scissors in air, b‐3) disassembly, b‐4) ultrasonic processing of the [3D‐SLISE layer]/[anode]/[Ti foil] in water; b‐5) Ti foil after processing; b‐6) suction filtration of cathode material dispersion. c‐1) Separation of LiCoO_2_‐rich powder (on filter) and 3D‐SLISE dispersion (filtrate), SEM/EDX images of the c‐2) filter surface, and c‐3) precipitate after drying the filtrate.

Our results confirm the feasibility of direct recycling of active materials. A 3D‐SLISE sheet on polyethylene terephthalate (PET) release film can be removed as a self‐supporting sheet (**Figure** **8**a‐1), which dissolves immediately when stirred in water (Figure 8a‐2; Movie S1, Supporting Information). This property allows for the direct recovery of active material from electrode scrap and defective batteries without its destruction, enabling a new recycling system without converting the material into a black mass. To illustrate safety, process suitability, and recyclability, a skeleton‐type battery (2.35 V) was fabricated using 3D‐SLISE as the electrolyte (Movie S2, Supporting Information). Connecting these in series could power a white LED (light‐up voltage >3.5 V) (Figure 8b‐1). The structure and characteristics of the skeleton battery are detailed in Figure S15 (Supporting Information), with one example shown in Figure S16 (blue curves, Supporting Information). Weak stack pressure (0.2 MPa) leads to insufficient interfacial contact and poor cycle characteristics. Increasing the stack pressure to 30 MPa stabilizes these characteristics (red curves). After a lighting test, a skeleton battery was cut with scissors in a laboratory (30% RH) (Figure 8b‐2) and separated into [electrolyte (3D‐SLISE) layer]/[cathode]/[Ti foil] and [anode]/[Al foil] (Figure 8b‐3). Scratching the [3D‐SLISE layer]/[cathode]/[Ti foil] with a spatula revealed no wet materials, confirming a solid‐based system. When soaked in water and treated with ultrasound, the cathode material dispersed immediately (Figure 8b‐4), leaving pure Ti foil (Figure 8b‐5). The water‐based dispersion of the cathode material was filtered through a membrane (Figure 8b‐6), separating the cathode active material into powder and electrolyte dispersion (3D‐SLISE) (Figure 8c‐1). The dried powder on the filter (Figure 8c‐2) was identified to be 10–20 µm Co‐containing particles—specifically LCO active material. Fluorine, a component of 3D‐SLISE, was detected throughout the surface of the dried filtrate, but no cobalt, originating from the cathode active material, was found (Figure 8c‐3). These results indicate that 3D‐SLISE is a rare material enabling direct recycling without the calcination process for binder burn‐out,^[^ ^41^ ^]^ thus contributing to reduced CO_2_ emissions. Incorporating direct recycling of active materials from electrode scrap, defective batteries, and aged batteries into the industry is expected to reduce CO_2_ emissions, secure resources, and lower raw material costs. Additionally, with the adoption of the Battery Passport system as an international standard, users can choose safe, reliable batteries with benefits like discounts, promoting the industry's green transformation. We anticipate the emergence of a new industrial chain focused on direct recycling technologies for active materials.

## 3 Conclusion

A novel quasi‐solid electrolyte with a 3D conduction pathway, born from the slime analogy, was found. Nonsintered quasi‐solid Li‐ion conductors (3D‐SLISE) comprising mechanochemically treated a‐Li_2_B_4_O_7_, LiFSI, and H_2_O were fabricated under atmospheric conditions without utilizing a glove box or dry room. The as‐prepared 3D‐SLISE slurry and an electrode slurry containing 3D‐SLISE were applied onto current‐collecting foils and naturally dried. 3D‐SLISE films with 7 wt.% of bound water showed an ionic conductivity of 2.5 mS cm^−1^ and a conduction activation energy of 0.25 eV in the temperature range from −20 to 80 °C. The electrochemical properties of the prepared 3D‐SLISE are attributed to the 3D network of novel interfacial structures generated by the binding of a‐Li_2_B_4_O_7_ active surface, FSI anions, and H_2_O molecules. 3D‐SLISE‐QSSBs comprising LCO cathode/3D‐SLISE layer/LTO anode showed a discharge voltage of 2.35 V and energy density (actual weight regulations in experiments) of 50 Wh kg^−1^ at 1C and 27 °C, discharged 160 mAh g^−1^ (on a LTO mass‐basis) at 3C and 27 °C, and maintained 92% of the discharge capacity over 400 cycles. Furthermore, the implementation of high‐capacity active materials (TNO) showed the potential for deployment in 100 Wh kg^−1^ class batteries.

Overall, this paper presents a LIB fabrication technology that does not require meticulous dew point control and heat drying, which could revolutionize the battery industry by facilitating significant improvements from the perspective of environmental safety and lowering the manufacturing costs of batteries. Although several issues require resolution, the proposed technology exhibits high potential for growth.

## 4 Experimental Section

### Sample Preparation

Each sample‐synthesis step was carried out under atmospheric conditions (25 °C and 30% RH). In this study, the composite matrix material (a‐Li_2_B_4_O_7_) was prepared by the mechanochemical milling (Fritsch P‐5, powder: ZrO_2_ beads (weight ratio) = 1: 40, treatment time = 45 h) of c‐Li_2_B_4_O_7_ (Fujifilm Wako Co.). The electrolyte slurry with a solid content of 70 wt.% was prepared by mixing a‐Li_2_B_4_O_7_, LiFSI (Fujifilm Wako Co.), 1.5 wt.% of CMC (DAICEL 1350, DAICEL Co.) and pure water (18.2 MΩ⋅cm), and dispersed using an ARE‐250 mixer (THINKY Co.). Cathode slurries with a solid concentration of 58.8 wt.% were prepared by mixing lithium cobalt (III) oxide (LiCoO_2_) powder (TOSHIMA Manufacturing Co.) with a median particle size of 12 µm, a‐Li_2_B_4_O_7_, LiFSI, carbon nanotube (CNT) (KJ SPECIALITY PAPER Co.), CMC, and pure water in the weight ratio of 48.3:4.1:4.6:1.0:0.8:41.2. Anode slurries with a solid concentration of 46.8 wt.% were prepared by mixing lithium titanate (Li_4_Ti_5_O_12_) powder (ISHIHARA SANGYO Co.) with a median particle size of 7 µm, a‐Li_2_B_4_O_7_, LiFSI, CNT, CMC, and pure water in the weight ratio of 34.8:4.7:5.1:1.9:0.3:53.2.

To fabricate 3D‐SLISE devices for conductivity measurements, the 3D‐SLISE slurry was first applied onto Al (50 µm thick) or Ti (20 µm thick) foils (Takeuchi Metal Foil & Powder Co.) using an automatic film applicator (BEVS1818, Allgood Co.). After natural drying, two pieces were punched out from the sheet with 10 mmϕ, and the 3D‐SLISE surfaces of each piece were placed facing each other to form the evaluation element. These elements were set in a uniaxial pressure fixture KP‐SolidCell (Hohsen Co.), pressed with a torque of 5 Nm (equivalent to an effective pressure of 30 MPa), heated at 80 °C for 2 h, and then cooled to 27 °C. The compact 300‐µm thick layer between the two metal foils was used for impedance testing.

For battery construction, cathode and anode slurries were applied onto Ti (20‐µm thick) and Al (50‐µm thick) foils, respectively. After naturally drying the cathode slurry for 30 min at 25 °C under 30% RH, the 3D‐SLISE slurry was applied (to form a multilayer). After natural drying, the 3D‐SLISE/cathode and anode sheets (comprising 16.8 mg cm^−2^ of LiCoO_2_ and 7.7 mg cm^−2^ of Li_4_Ti_5_O_12_, respectively) were punched to dimensions of 10 and 8 mmϕ, respectively. Subsequently, the electrodes were joined, set in a uniaxial pressure fixture KP‐SolidCell (Hohsen Co.), pressed with a torque of 5 Nm (equivalent to 30 MPa), heated at 80 °C for 2 h, and then cooled to 27 °C. Cells comprising an 80‐µm thick cathode, 60‐µm thick 3D‐SLISE layer, and 100‐µm thick anode were used for impedance and battery charge–discharge testing.

TiNb_2_O_7_ (TNO) crystal powder, a high‐capacity anode active material, was synthesized by mixing rutile‐type TiO_2_ (99.99%, RARE METALIC Co.) and Nb_2_O_5_ (99.99%, RARE METALIC Co.) with 15% more Nb from stoichiometry and sintering at 1250 °C for 12 h. The preparation and application of the TNO‐based anode slurry were carried out in the same way as for LTO. 3D‐SLISE was applied to the TNO anode coating formed on the Al foil and allowed to dry naturally at 25 °C and 30% RH. The TNO‐based anode/Al sheet with a TNO weight of 8.4 mg cm^−1^ was punched out to 10 mmϕ, and the LCO cathode/Ti sheet with a weight of 14.1 mg cm^−2^ was punched out to 8 mmϕ, and the battery was made using the method described above.

### Mass Determination of Active Material

The cathode/collector sample or the anode/collector sample was punched out to 10 mmϕ, immersed in pure water, and the electrode layer was scraped off into the water using a spatula. The current collector was removed from the water and the suspension of the electrode components was suction filtered through an 1825‐090 GF/F (9.0 cm) glass microfiber filter (Whatman Co., particle‐retaining performance: 0.7 µm). The filter paper was previously dried at 120 °C for at least 3 h and weighed. After filtration, the filter paper on which the active‐material‐based powder was collected was dried at 120 °C for at least 3 h and then weighed. The weight difference between the filter paper that collected the powder and the filter paper itself was used as the amount of active material.

### Particle Size Distribution and Bulk Modulus Analysis

A suspension of c‐Li_2_B_4_O_7_ particles (0.6 wt.%) in pure water (18.2 MΩ⋅cm) was used to record ultrasonic attenuation spectra (10–70 MHz). Measurements were conducted in a water tank with an incident transducer, sample cell, and detection transducer aligned linearly, utilizing a polystyrene cuvette (optical path length: 10 mm, thickness: 2.3 mm) as the sample cell. The elastic modulus of c‐Li_2_B_4_O_7_ particles was determined by fitting to the scattering attenuation theory equation (Equation 1):^[^ ^42^, ^43^ ^]^

(1) $$\alpha \left(\omega \right)=\alpha_{0}-\sum_{i}\frac{2\pi N_{i}}{k_{1}^{2}}Re\left(\sum_{n=0}^{\infty }\left(2n+1\right)A_{n,i}\left(a_{i},\rho_{1},\rho_{2},c_{1},c_{l2},c_{s2},\sigma_{2}\right)\right)$$

Here, *α*(*ω*) is the decay rate of ultrasound at frequency *ω*, *α*_0_ is the decay rate caused by something other than particles, *k*_1_ is the scattering vector in the medium, *N*_1_ is the number frequency of particle *i*, *A_n,i_* is the nth‐order scattering coefficient of particle *i*, *a_i_* is the radius of particle *i*, *ρ*_1_ is the specific gravity of the medium, *ρ*_2_ is the specific gravity of the particle, *c*_1_ is the speed of sound in the medium, *c_l_*_2_ is the longitudinal sound speed in the particle, *c_s_*_2_ is the transverse sound speed in the particle, and *σ*_2_ is Poisson's ratio of the particle. The particle size distribution of c‐Li_2_B_4_O_7_ was determined using image analysis, and the results were used as input values for fitting. The density and Poisson's ratio of the c‐Li_2_B_4_O_7_ particles were 2.45 g mL^−1^ and 0.12, respectively. A suspension of a‐Li_2_B_4_O_7_ (1.2 wt.%) in pure water was used for experimentation, and the elastic modulus of a‐Li_2_B_4_O_7_ particles was determined by the procedure described above.

### Film Thickness Measurement

The film thickness was measured using a laser displacement meter (KEYENCE CL‐P015G). To measure the film thickness profile of a naturally dried film fixed with a vacuum chuck, the sensor head of the laser displacement meter was scanned perpendicular to the application direction. A stepping motor (D220, SURUGA SEIKI) was used to scan the sensor head. The difference between 3D‐SLISE‐applied and non‐applied Al foils was considered to be the 3D‐SLISE‐film thickness.

### Compositional Analysis

To quantify Li, B, and Na, a 10 mg sample was ashed in a Teflon container with 5 mL of 70% nitric acid (MultiWave 3000, Anton Paar GmbH) and diluted to 50 mL with pure water (*n* = 3). The solution was analyzed using atomic emission spectrometry (ICPE‐9820, SHIMADZU Co.). To determine the amounts of F and S in the sample, the sample (1 mg) and WO_3_ (20 mg) were weighed in a magnetic boat (pre‐baked at 1000 °C for 30 min) and stirred until homogeneous (*n* = 3). A mullite tube containing this sample mixture was placed in an AQF‐2100H (outlet temperature: 1100 °C, inlet temperature: 1100 °C), and absorbed into a hydrogen peroxide solution (300 ppm). Subsequently, a constant volume was prepared according to the “High” setting of the constant‐volume sensor (≈16 mL, calculated from the intensity), and 100 µL of this solution was injected into the ion‐chromatography unit. Calibration curves constructed using 0, 5, 10, and 20 ppm of F and S standards were used to calculate the concentrations of F and S in the sample.

### Structural Analysis

Powder X‐ray diffraction patterns were recorded on a BRUKER D2 PHASER (Cu source (λ = 1.54 Å), 30 kV, 10 mA). A Rigaku SmartLab (Mo source (λ = 0.71 Å), 60 kV, 150 mA) was used for X‐ray total‐scattering measurements. Prior to analysis, the samples were packed into borosilicate capillaries (1.5 mmϕ) under 11% RH and sealed. The SmartLab Studio II software (Rigaku) was used for pre‐processing (including background‐intensity analysis (using blank capillaries), Compton scattering and X‐ray polarization corrections, and powder diffraction file (PDF) analysis. The structure factor, S(Q), was calculated using a Q range of 0.46–17.26 Å^−1^ and used to generate a S(Q)‐based reduced PDF.

### Morphological Analysis

A Bruker Dimension ICON and a scanning microwave impedance microscope (sMIM) with a Primenano ScanWave Pro were used for atomic force microscopy (AFM) under nitrogen. Prior to surface measurements, the sample was fixed onto a metal plate using conductive tape (for continuity with the sample table). A Bruker sMIM‐150 (material: TiW, f = 75 kHz, k = 8 N m^−1^) was used as the probe for measurements in the PeakForce sMIM mode (scan speed: 0.5 Hz, pixel size: 256 × 256).

The voltage derivative of the capacitance (dC/dV) signal was obtained using sMIM data recorded under the previously described conditions with the application of an AC bias (175 kHz, 1 V) on the sample. Here, p‐ and n‐type metal oxide semiconductor standard samples were pre‐measured to determine the phase and positive/negative correspondence between the carriers.

The battery cross‐section morphology was investigated through scanning electron microscopy (SEM; JEOL JCM‐7000). Reflection electron microscopy images were acquired at an acceleration voltage of 15.0 kV.

### Water Content Analysis

A Mitsubishi Analytec CA‐200 and a heating drive unit (VA‐230) were used for water content (C_w_) determination of 3D‐SLISE by the Karl Fischer titration method. The temperature of the experimental setup was maintained at 130 °C. Aquamicron AX and Aquamicron CXU were used as anode and cathode solutions, respectively. The samples were prepared in screw‐type vials manufactured by Nitto Seiko Analytics.

The water content of 3D‐SLISE in actual batteries (≠ battery water content) was evaluated by combining the Karl Fischer method and mass determination of active materials: the weight (W_total_) and water content (W_H2O_) of a 10 mmϕ battery were measured, the amount of active materials in the cathode and anode were determined (W_LCO_, W_LTO_), and the 3D‐SLISE water content (C_w_) in the battery was calculated using Equation (2).

(2) $$C_{w}=W_{H2O}/\left(W_{\mathrm{total}}-W_{\mathrm{LCO}}-W_{\mathrm{LTO}}\right)\times 100\;\left[\mathrm{wt}.\%\right]$$

### Computational Analysis

The Vienna Ab‐initio Simulation Package was used for DFT calculations of the electronic structure.^[^ ^44^ ^]^ The Perdew–Burke–Ernzerhof generalized gradient‐corrected exchange functional was used to evaluate the total energies with a supercell approach at the Γ point.^[^ ^45^ ^]^ DFT‐D3 with Beck‐Johnson damping was used to account for van der Waals interactions.^[^ ^46^ ^]^ A Nose thermostat at a controlled temperature was used for DFT–MD simulations with a plane energy of 500 eV and a time step of 1 fs.^[^ ^47^, ^48^ ^]^ The amorphous structure of Li_2_B_4_O_7_ (Li_16_B_32_O_56_) was generated from its crystalline structure using the melt‐quench method. MD calculations were carried out every 200 K for 1 ps while increasing the temperature from 400 to 2600 K, followed by cooling to 400 K. The 3D‐SLISE complex was modeled using two supercells. The supercells 8Li_2_B_4_O_7_–4LiFSI–26H_2_O and 8Li_2_B_4_O_7_–3LiFSI–29H_2_O with dimensions of 10.16 × 10.16 × 20.32 Å^3^ were constructed and used for DFT–MD simulations. MD calculations of equilibrium relaxation at 300 K were carried out after MD calculations at 700 K and 4 ps (to accelerate structural relaxation). After equilibration, the statistical averages were calculated from the trajectories of at least 2 ps.

Machine learning potential molecular dynamics (MLP‐MD) simulations were conducted using the momentum‐tensor descriptor.^[^ ^49^ ^]^ The time step of MD was set to 0.5 fs, and the temperature was fixed at 300 K. MD simulations were performed using the LAMMPS package.^[^ ^50^ ^]^ The system sizes for the calculations were 80LiFSI‐160H_2_O, 108LiFSI‐300H_2_O, and 54LiFSI‐300H_2_O for the 28, 20, and 10 mol kg^−1^ LiFSI‐H_2_O eutectic systems, respectively.

### Electrochemical Measurements

An *N*‐methyl‐2‐pyrrolidone slurry of LiFePO_4_ (LFP), acetylene black, and polyvinylidene fluoride in the weight ratio of 7:2:1 was applied onto a Ti foil (20‐µm thick). After solvent drying, this electrode was used as the WE to partially delithiate LFP by voltage sweeping from the open‐circuit potential to 0.28 V (vs Ag/AgCl) using a lithium bis(trifluoromethanesulfonyl)imide (LiTFSI) solution (1.2 mol kg^−1^) (scan rate: 0.5 mV s^−1^, CE: LiMn_2_O_4_, reference electrode (RE): Ag/AgCl). The LFP electrode showed an acceptably stable electrode potential (vs Ag/AgCl, measured in a LiTFSI solution (1.2 mol kg^−1^)) as an RE. Considering the known electrode potentials of Ag/AgCl (0.199 V vs SHE) and Li^+^/Li (−3.045 V vs SHE), the potential of the LFP RE was estimated to be 3.44 V versus Li^+^/ Li.

The oxidation (and reduction) potentials of the electrolyte were evaluated by linear sweep voltammetry using Ti (or Al), Li_4_Ti_5_O_12_ (or LiCoO_2_), and Li_(1‐x)_FePO_4_ as the WE, CE, and RE, respectively, at a scan rate of 0.2 mV s^−1^.

The redox reactions of the active materials LiCoO_2_ and Li_4_Ti_5_O_12_ were investigated using C‐V with LiCoO_2_ (or Li_4_Ti_5_O_12_) as the WE, Li_4_Ti_5_O_12_ (or LiCoO_2_) as the CE, and Li_(1−x)_FePO_4_ as the RE, at a scan rate of 0.2 mV s^−1^. All measurements were conducted at 27 °C using a BAS ALS832D potentiostat.

### Li^+^ Transportation Analysis

The diffusion coefficients of Li^+^ and FSI^−^ were analyzed using the pulsed field gradient NMR method, and the transport number of Li^+^ was evaluated. 3D‐SLISE powders with different water contents (weight ratio: LBO/LiFSI/CMC/H_2_O = 47/51/2/x) (water content C_w_: 7, 10 wt.%) were prepared (by drying and scraping the applied film in a humidity‐controlled glove box), and placed in a symmetric micro‐sample tube (Shigemi BMS‐005J). The sample thickness was adjusted to 5 mm or less to ensure that the entire sample was subjected to a uniform magnetic field gradient. To maintain airtightness, the sample was sealed with epoxy adhesive (Bond Quick 5, manufactured by Konnishi Co., Ltd.) and removed from the glove box.

For the measurements, a JEOL 400 MHz NMR spectrometer (ECX‐400) with a GR probe (maximum magnetic field gradient strength: 1376 G cm^−1^) was used. The bipolar‐pulse pair stimulated echo method with longitudinal eddy current delay was used to evaluate the diffusion coefficient.^[^ ^51^ ^]^ The gradient recovery and longitudinal eddy current delay were measured under the conditions τg = 0.1 ms and Te = 50 ms, respectively. Sinusoidally shaped gradient pulses were used to suppress eddy currents. The main measurement parameters are shown in **Table** **4**. The diffusion coefficient was calculated by regression of the dependence of the NMR signal on the magnetic field gradient strength using the following theoretical Equations (3) and (4). Here, I(k) is the NMR signal intensity, D is the diffusion coefficient, γ is the gyromagnetic ratio, and g is the magnetic field gradient strength.

**Table 4:** Key measurement parameters for pulsed field gradient NMR measurements.

Nuclear spieces	^7^Li	^19^F
δ (ms)	1	1
Δ (ms)	100	50
Observation center (ppm)	0	40
Data points	512	256
Acquisition time (ms)	26	5.4
Waiting time (s)	5	2
Number of dummy scans	4	4
Accumulation	8–32	16–32

(3) $$I\left(k\right)\propto \exp\left(-\mathrm{kD}\right)$$

(4) $$k=\frac{4}{\pi^{2}}\gamma^{2}g^{2}\;\delta^{2}\left(\Delta -\frac{2\delta }{3\pi }-\frac{\tau_{g}}{2}\right)$$

### AC Impedance Measurements

The AC impedance profiles of 3D‐SLISE were measured using an SP‐300 (Bio‐Logic Science Instruments Co.) under uniaxial pressure of 30 MPa with the sample placed in KP‐SolidCell (Hohsen Co.) (configuration: [aluminum or titanium foil]/[3D‐SLISE layer]/[aluminum or titanium foil]). The AC impedance of 3D‐SLISE was investigated on an SP‐300 (Bio‐Logic Science Instruments) instrument under a uniaxial stack pressure of 30 MPa after setting the sample element using KP‐SolidCell (Hohsen Co.) in the [Al or Ti foil]/[3D‐SLISE layer]/[Al or Ti foil] configuration. The battery configuration was evaluated under a uniaxial stack pressure of 30 MPa using the [Ti current‐collecting foil]/[cathode]/[3D‐SLISE layer]/[anode]/[Al current‐collecting foil] configuration.

### Charge–Discharge Analysis

For the charge–discharge evaluation of the 3D‐SLISE batteries, sample elements set in KP‐SolidCell (Hohsen Co.) (configuration: [Ti current‐collecting foil]/[cathode]/[3D‐SLISE layer]/[anode]/[Al current‐collecting foil configuration]) were analyzed using a Scribner Associates 580 instrument under a uniaxial stack pressure of 30 MPa.

### Chemical State Analysis of the Anode

After the cycle test, the battery was disassembled in a glove box, and the Al current collector foil and anode were removed to expose the anode as the sample. This sample was sealed in a tube and transferred to the X‐ray photoelectron spectroscopy (XPS) chamber. XPS measurements were conducted without air exposure on a VersaProbe II (ULBAC‐PHI Co.) using monochromatic Al‐Kα excitation light (parameters: 1486.6 eV, 25 W, 15 kV, and 100 µmϕ), with a measurement area of 100 µm × 100 µm and photoelectron extraction angle of 45°.

### Quantitative Analysis of LiFSI in Batteries

The sample was placed in a 20 mL screw‐top vial for headspace (HS), pre‐weighed in a nitrogen‐purged glove box (GB). The total sample weight was determined by weighing the sealed vial post‐removal. Methanol (10 mL) was added, and the mixture was ultrasonically extracted until the electrode was detached and dispersed. The detached electrode/metal foil was collected and weighed, and its weight was subtracted from the total sample weight to determine the weight of the cathode/3D‐SLISE layer/anode. The extraction solution was centrifuged (20 000 g for 5 min), the supernatant was diluted 10–25 000‐fold with ultrapure water, and filtered through a 0.45 µm filter before measurement. The calibration curve solution was prepared similarly by dividing an appropriate amount into an HS vial in the GB; the vial was then sealed and weighed, and finally, its content was diluted with ultrapure water to achieve a constant volume. Instrument: Shimadzu Nexera X2 /LCMS‐2020, column: CAPCELL PAK C18 UG120, 5 µm (2.0 × 50 mm), column oven temperature: 40 °C, eluent A: 10 mM CH_3_COONH_4_/H_2_O, eluent B: 10 mm CH_3_COONH_4_/CH_3_CN:H_2_O = 90:10, flow rate: 0.2 mL min^−1^, injection volume: 10 µL, gradient: B 10% (0 min) to B 100% (30–40 min) with a 15 min post‐run, detector: PDA, ESI‐MS (scan, positive/negative ion mode, m/z 100–1000, quantitative SIM m/z 179.8, absolute calibration method).

### Gas Analysis

After the charge–discharge test, the measurement jig was placed in a heat‐sealed aluminum bag, which was then shaken to evenly distribute the gas. A gas‐tight syringe was used to collect a sample for measurement. The detailed conditions are as follows. Instrument: Shimadzu QP‐2030, injection volume: 0.2 mL (gas‐tight syringe), inlet temperature: 250 °C, column: MICROPACKED‐ST 1.0 mm ID × 2.0 m, carrier gas: He (226.8 kPa–2.5 min → 15.2 kPa min^−1^ → 352.2 kPa–4.25 min), injection method: split 4:1, oven temperature: 35 °C–2.5 min → 20 °C/min → 200 °C–4.5 min, detector: BID (He 50 mL min^−1^, 280 °C), quantitative method: absolute calibration method (H_2_: 25–600 µL L^−1^, CO: 2.5–60 µL L^−1^). The gas concentration was determined using a calibration curve. Subsequently, the total volume of the Al bag was measured by water displacement, and the gas amount was calculated by multiplying the concentration by the volume.

### Fabrication, Evaluation, Disassembly of Skeleton Batteries, and Demonstration of Direct Recovery of Cathode Active Material

The procedure for fabricating a skeleton battery in the air (25 °C, 30% RH) is illustrated in Figure S15 (Supporting Information). A 3D‐SLISE layer/cathode layer/Ti sheet was cut to 6 cm × 2.8 cm, and an Al tab with a sealant was attached to the back edge of the Ti using silver paste. Similarly, the anode layer/Al sheet was cut to 6 cm × 2.2 cm, and an Al tab with a sealant was attached to the back edge of the Al using silver paste. These parts were stacked with the 3D‐SLISE layer and anode layer in contact over an area of 4.5 cm × 2.2 cm, with Al tabs facing outward. This assembly was sandwiched between 8 cm × 4 cm PACOPADs (ESE Industries Co.) and hot‐pressed for 5 min at 80 °C and 5 MPa using a hot press machine (Leabdio Co.). PACOPADs ensured uniform hot pressing. After hot pressing, clean gas barrier films (Universal Co.) were placed on top and bottom and vacuum packed using a tabletop vacuum packaging machine (TAKATO TECHNICA Co. VP‐300). The assembly was then sandwiched between 3‐mm‐thick acrylic boards and held with clips. The confining pressure was estimated at 0.2 MPa using a pressure measurement film (Prescale LLLW, Fujifilm Co.).

The effective area was 5.1 cm^2^, with 18 mg of anode active material (Li_4_Ti_5_O_12_). The test used excess positive electrode active material. Four skeleton batteries were fabricated and charged after three charge/discharge cycles at 0.3 C and room temperature under a stack pressure of 0.2 MPa. The parallel pairs were connected in series and tested for white LED lighting for 4 h (Figure 5b‐1). Afterward, the stack pressure of 0.2 MPa was replaced with a pressure of 30 MPa using a self‐made jig, and charging/discharging was evaluated at room temperature and 1 C rate. The 30 MPa pressure was confirmed using a pressure measurement film (Prescale MS, Fujifilm Co.).

The one skeleton battery used for LED lighting was cut in an atmospheric environment. The laminate was removed, and the top and bottom electrodes were separated into [3D‐SLISE layer]/[cathode layer]/[Ti foil] and [anode layer]/[Al foil]. The [3D‐SLISE layer]/[cathode layer]/[Ti foil] was soaked in water, ultrasonically treated for 1 min while held with tweezers, and shaken. After treatment, the Ti foil was removed, and the remaining cathode material dispersion was filtered using a membrane filter (Whatman Co. GF/F glass microfiber filter 1825‐090, particle‐retaining performance: 0.7 µm). The filter paper surface and filtrate were observed using SEM/EDX.

## Conflict of Interest

The authors declare no conflict of interest.

## Author Contributions

Y.S., Y.O., and S.Y. proposed the concept. Y.S., K.W., K.S., and R.S. designed the experiments. Y.S., K.W., and K.S. developed the 3D‐SLISE system. Y.S., K.W., K.S., R.S., and S.Y. analyzed the structures and basic physicochemical and electrochemical properties. Y.S. and K.W. designed the electrochemical cell and conducted cycling tests. Y.O. designed and carried out theoretical calculations. All authors contributed to the discussion. Y.S. wrote the manuscript. S.Y. supervised the study.

## Supporting information



Supporting Information

Supplemental Movie 1

Supplemental Movie 2

## Data Availability

The data that support the findings of this study are available from the corresponding author upon reasonable request.
