# Supplementary material for: Borate‐Water‐Based 3D‐Slime Interface Quasi‐Solid Electrolytes for Li‐ion Batteries
Source: Adv Mater. 2025 Jul 9;37(41):2505649. doi: 10.1002/adma.202505649 (PMC12531718; doi:10.1002/adma.202505649)
Supplement: Supplementary file 1 — Supporting Information [file ADMA-37-2505649-s001.pdf]

ADVANCED MATERIALS

Supporting Information

for *Adv. Mater.*, DOI 10.1002/adma.202505649

Borate-Water-Based 3D-Slime Interface Quasi-Solid Electrolytes for Li-ion Batteries

Yosuke Shiratori, Kenta Watanabe, Kengo Saito, Ryota Sato, Yukihiro Okuno and Shintaro Yasui**

Supporting Information

Borate-Water-Based 3D-Slime-Interface Quasi-Solid Electrolytes for Li-ion Batteries

Yosuke Shiratori, Kenta Watanabe, Kengo Saito, Ryota Sato, Yukihiro Okuno, and Shintaro Yasui

The PDF file includes:

Figures S1 to S16

Tables S1 to S3

Experimental Section

References

Other Supporting Material for this manuscript includes the following:

Movies 1 to 2

Table S1. Characteristics of aqueous liquid electrolytes and aqueous quasi-solid electrolytes

Electrolyte		Process				Electrochemical properties		Battery properties		
Composition	Type	Separator	Cathode binder	Electrolyte injection	Direct multilayer application 5)	Ionic conductivity (mS cm^{-1})	Potential window (V)	Cell voltage (V)	Energy density (Wh kg^{-1})	Cycle life 6)
Water-in-salt [1]	CL 1)	Required	PTFE 3)	Required	–	~ 10	1.9 – 4.9	2.30	80 – 100	1000
Hydrate-melt [2]	CL	Required	PVDF 4)	Required	–	~ 3	1.3 – 5.1	3.10	130	Several hundred
Eutectic gel [3]	QS 2)	Required	PVDF	Required	–	~ 1	0.7 – 5.0	2.35	–	500
**3D-SLISE** This work	QS	Not required	CMC (Water soluble)	Not required	Demonstrated in this paper	~ 2	1.5 – 4.5* (*at least)	2.35	50 – 100	400 7) – 2000 8)

1) CL: concentrated liquid, 2) QS: quasi-solid, 3) PTFE: poly(tetrafluoroethylene), 4) PVDF: polyvinylidene difluoride, 5) All solid-state battery process itself, 6) At high rate: > 3C, 7) At present (this paper), 8) Improved data will be reported in elsewhere (in preparation).

Table S2. Composition dependence and optimization of the slurry properties.

	Gel, slurry, liquid	Composition (wt%) / (molar ratio)				Appearance
		a-$Li_2B_4O_7$	LiFSI	CMC	H_2O	
1	a-$Li_2B_4O_7$<br>+ H_2O	51.9<br>1.0	0<br>–	0<br>–	48.1<br>8.7	Gel 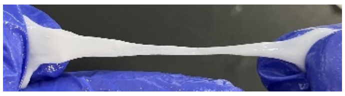 Pelletizing after drying 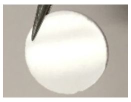
2	a-$Li_2B_4O_7$<br>+ CMC<br>+ H_2O	50.9<br>1.0	0<br>–	2.0<br>–	47.1<br>8.7	Gel 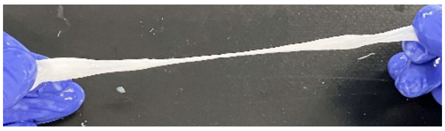
3	a-$Li_2B_4O_7$<br>+ LiFSI<br>+ H_2O	33.0<br>1.0	36.4<br>1.0	0<br>–	30.6<br>8.7	Low viscosity / poor dispersion 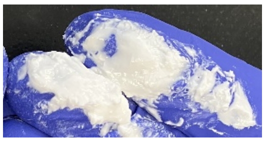
4	Suboptimal<br>3D-SLISE*<br>slurry	38.4<br>1.0	42.3<br>1.0	1.5<br>–	17.8<br>4.4	Jelly-like<br>Low viscosity<br>Well-dispersed 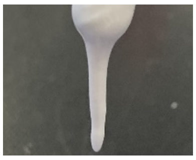
5	**3D-SLISE***<br>**Type S**<br>**Slurry**	32.6<br>1.0	35.9<br>1.0	1.3<br>–	30.2<br>8.7	Jelly-like<br>Higher viscosity<br>Well-dispersed<br>Application conformity 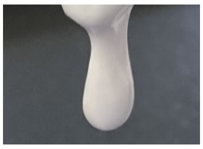
6	LiFSI<br>+ CMC<br>+ H_2O	0<br>–	53.3<br>1.0	1.9<br>–	44.8<br>8.7	Syrupy liquid 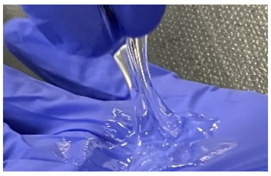
7	LiFSI<br>+ CMC<br>+ H_2O	0<br>–	84.6<br>1.0	3.1<br>–	12.3<br>1.5	Viscous liquid 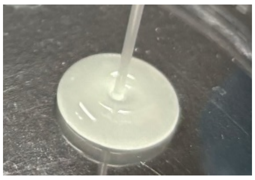
8	LiFSI<br>+ H_2O	0<br>–	54.3<br>1.0	0<br>–	45.7<br>8.7	Liquid 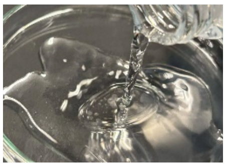
9	Reference<br>Hydrate-melt*<br>[2]	0<br>–	LiTFSI: 56.9<br>2.3<br>LiBETI: 32.9<br>1.0	0<br>–	10.2<br>6.7	Liquid 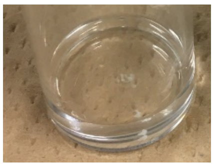

* Battery workable.

Table S2-1 shows the properties of various compositions (gel, slurry, liquid) before drying. The formation of a gel-like substance by the reaction of $Li_2B_4O_7$ with water has been reported in the previous study.[4] Although it depends on factors such as molecular concentration, temperature, and pH, the formation of polyborate species from boric acid in aqueous solution generally undergoes the reaction described by the following equation (1):

$$xB(OH)_3 + (y - z)H_2O \rightarrow B_xO_z(OH)^{y-}_{3x+y-2z} + yH^+ \quad (1)$$

,where x, y, z are variables representing the number of atoms or molecules participating in the reaction.[5]

Our slurry (3D-SLISE slurry) contains CMC. The hydroxyl groups of CMC in this system may promote cross-linking and gelation by reacting with boric acid. This process is similar to the reaction mechanism of traditional “slime” formulations.[6] These polyborate species and cross-linked structures are incorporated into the conducting interface of 3D-SLISE and may contribute to the transition from a gel-like state to a solid-like elasticity upon drying.

When a-$Li_2B_4O_7$ and water are mixed, a gel is formed (Table S2-1), and the same is true when CMC is added (Table S2-2). In the case of mixing a-$Li_2B_4O_7$, LiFSI, and water, a low-viscosity suspension (poor dispersion) is formed (Table S2-3), however, when CMC is added, the dispersion improves remarkably to a jelly-like state (Table S2-4), and furthermore, by adjusting the water content, a 3D-SLISE slurry with a viscosity suitable for application is obtained (Table S2-5). Considering from the LiFSI solution side (Table S2-8), adding CMC and adjusting the amount of water results in a viscous liquid (Table S2-7) and then a syrupy, highly viscous liquid (Table S2-6). The 3D-SLISE slurry is located between a-$Li_2B_4O_7$ gel (Table S2-1) and an aqueous solution of LiFSI (Table S2-8) and has properties suitable for application due to the effects of a-$Li_2B_4O_7$ and CMC. After the 3D-SLISE slurry is applied, it functions as a quasi-solid-state electrolyte by removing excess water that interferes with battery operation.

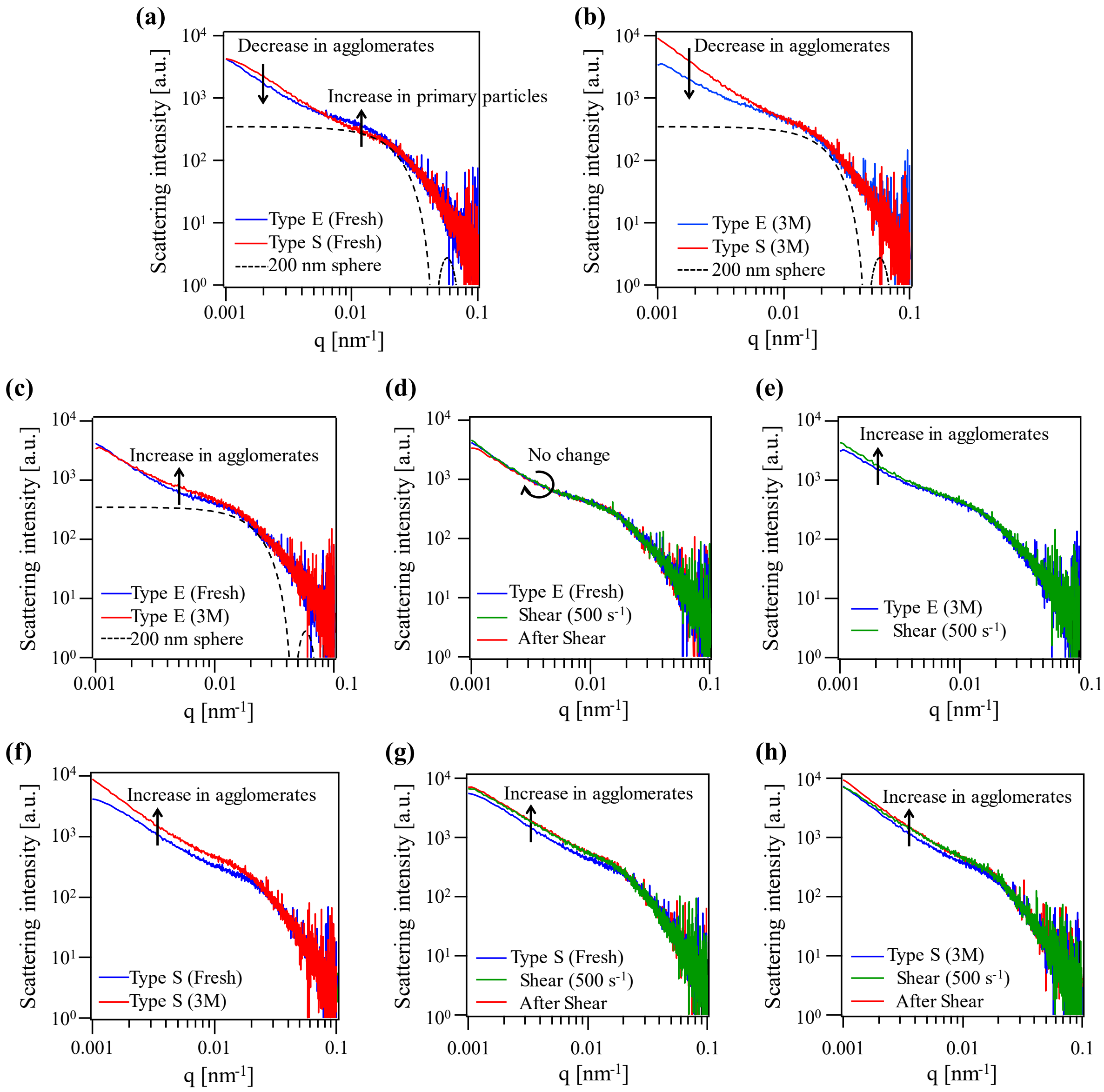

Figure S1. Ultra-small-angle X-ray scattering (USAXS) profiles of the 3D-SLISE slurries (Type S vs Type E), a) fresh samples, b) samples after 3 months refrigerated storage, c) fresh vs. after 3 months refrigerated storage (Type E), d) effect of shearing (Type E, fresh), e) effect of shearing (Type E, after 3 months refrigerated storage), f) fresh vs. after 3 months refrigerated storage (Type S), g) effect of shearing (Type S, fresh), h) effect of shearing (Type S, after 3 months refrigerated storage).

Figure S1a and S1b shows USAXS profiles obtained immediately after preparation (fresh) and after 3 months of refrigerated storage for Type S and Type E slurries, which reflect the

aggregate diameter. Type E was better dispersed than Type S in fresh and 3-month refrigerated storage slurries. Type E exhibited slight agglomeration after 3 months of storage (Figure S1c). However, there was no change in the dispersion state even after shearing, indicating high dispersion stability (Figure S1d). On the other hand, after 3 months of storage, agglomeration occurred when shearing was applied (Figure S1e). In Type S, the progress of agglomeration after 3 months of storage was more pronounced than in Type E (Figure S1f), and agglomeration occurred under shearing even in fresh samples (Figure S1g), indicating that the dispersion stability was low.

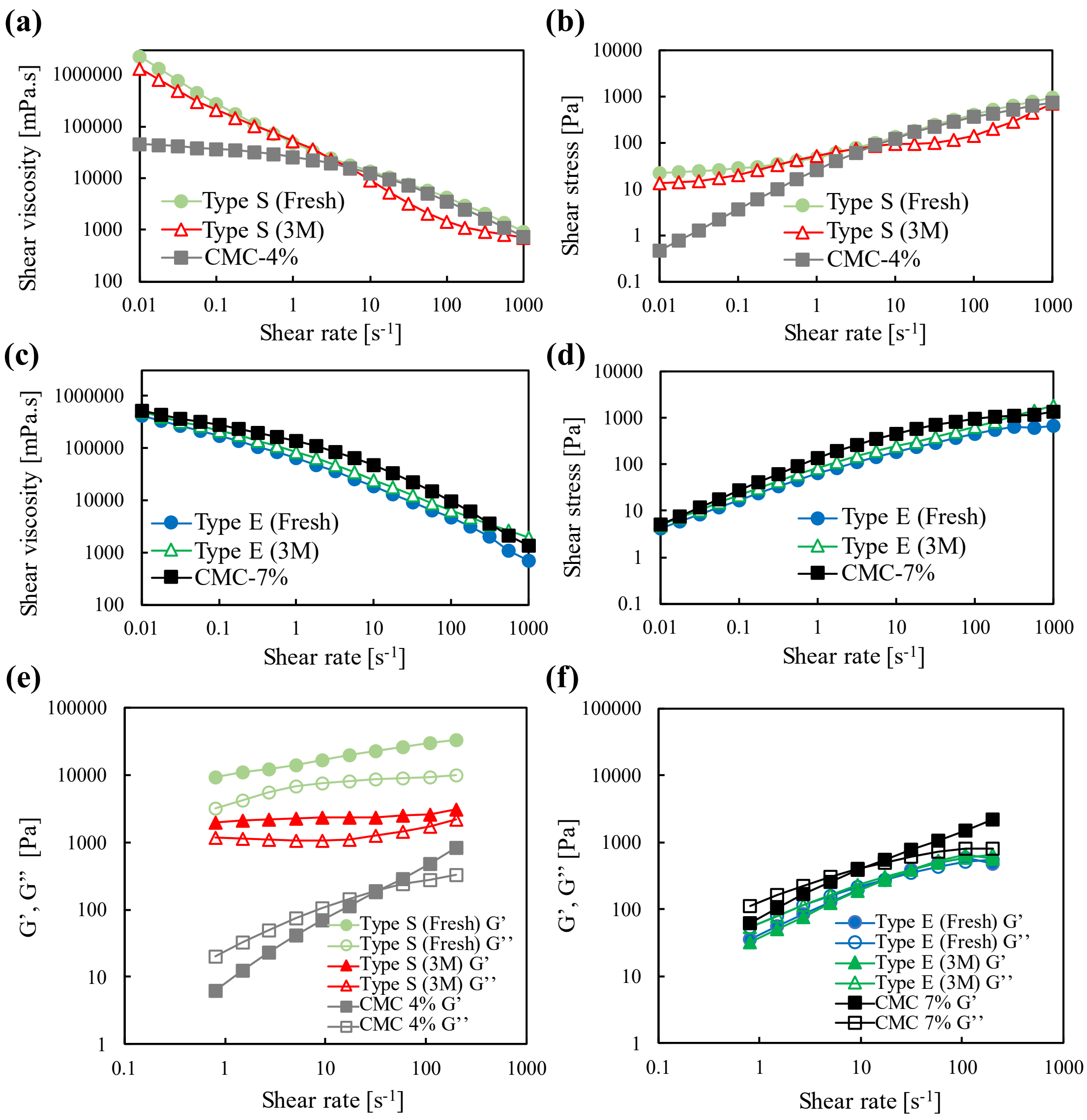

Figure S2. a), b) Shear viscosity and shear stress of Type S slurry and 4 wt% CMC solution under steady flow. c), d) Shear viscosity and shear stress of Type E slurry and 7 wt% CMC solution under steady flow. Frequency dependence of storage modulus (G') and loss modulus (G''), e) Type S slurry, f) Type E slurry.

Rheological analysis was performed on slurries Type S and Type E to understand the structure of the slurry, which is the structural origin of the electrolyte (3D-SLISE) (**Figure S2**). CMC solutions simulating the ideal dispersion state (4 wt% and 7 wt%, respectively) were also analyzed. In Type S (Figure S2a, S2b), the viscosity was high at low velocity and stress remained even at zero velocity, suggesting the formation of a solid-like structure. After 3

months of refrigerated storage, the dispersion became low viscosity, possibly due to changes in CMC adsorption ratio or molecular weight. Type E exhibited low viscosity variation with shear rate and time, and had properties similar to CMC solutions (Figure S2c, S2d), indicating that the dispersion state was close to ideal. It was considered that the particle dispersion was stably maintained by CMC adsorption. Figure S2e, S2f show the frequency dependence of the storage and loss moduli of the slurry; the elastic modulus (G') of Type S is significantly higher than that of Type E and did not relax at low frequencies. After 3 months of refrigerated storage, the elastic modulus decreased to less than 1/5 (Figure S2e). These results suggest that a persistent network (particle aggregation) is formed in Type S. In Type E, G' was lower than that of the CMC solution and decreased (relaxed) at lower frequencies (Figure S2f), suggesting that the surface adsorption of CMC kept the particle dispersion stable. The above dynamic viscoelasticity is similar to the properties of borax-PVA hydrogels (slime-like).[7]

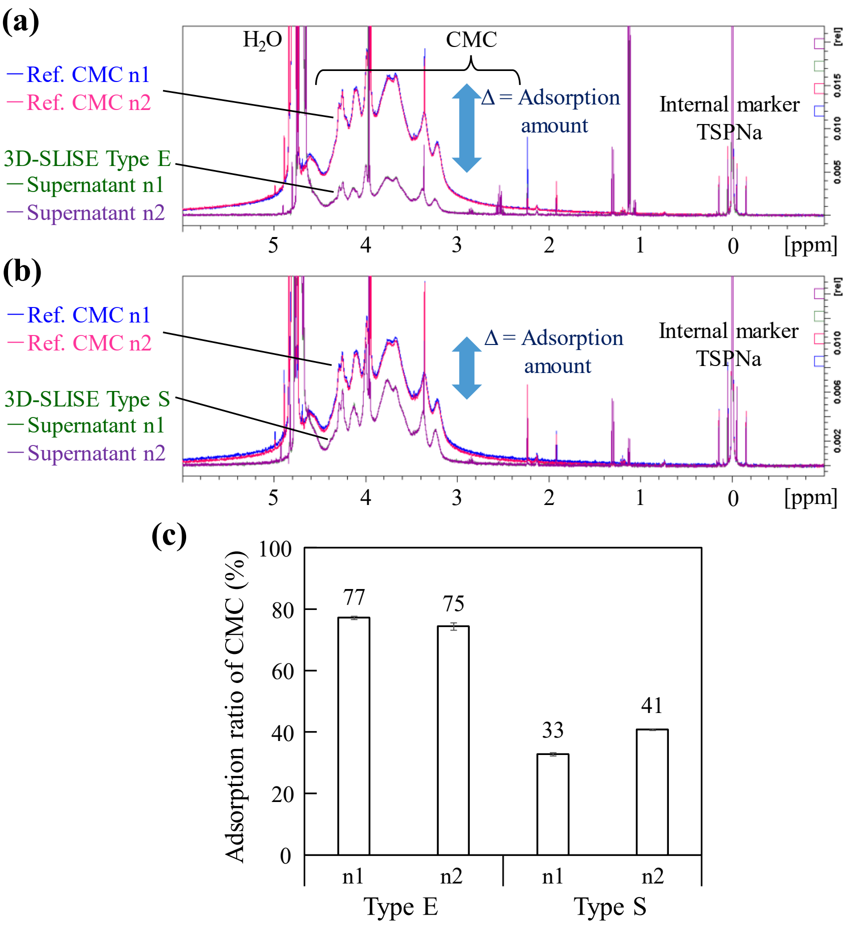

Figure S3. ^{1}H-NMR spectra of supernatant liquids obtained by centrifugation of 3D-SLISE slurries prepared with D_2O, a) Type E, b) Type S, and slurry prescription concentration equivalent CMC solutions. c) Adsorption ratio of free CMC in Type S and Type E slurries estimated from ^{1}H-NMR measurements.

The adsorption ratio of CMC in Type E and Type S slurries was analyzed by ^{1}H-NMR (**Figure S3a–S3c**). The CMC adsorption ratio was found to be 76% and 37%, respectively, indicating that the superior dispersibility of Type E over Type S is mainly due to the adsorption of CMC.

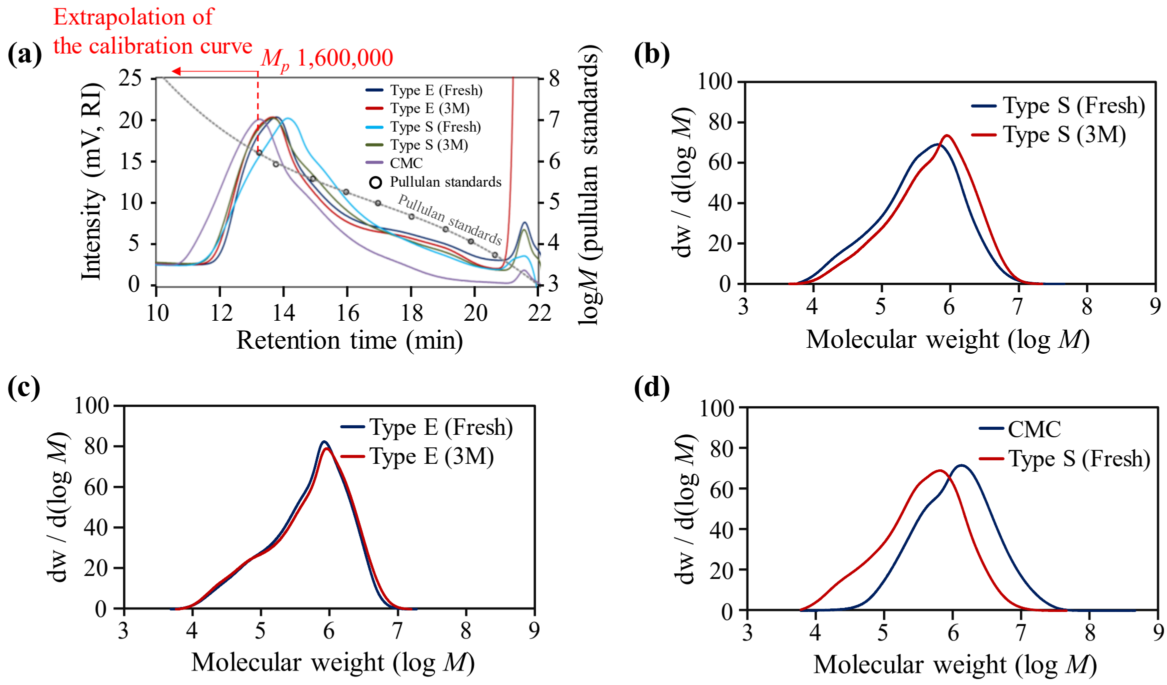

Figure S4. a) GPC chromatogram and pullulan calibration curve of CMC released from 3D-SLISE slurries (Type S, Type E). Molecular weight distribution of CMC, b) Type S slurry freshly prepared and after 3 months refrigerated storage, c) Type E slurry freshly prepared and after 3 months refrigerated storage, d) CMC standard and free CMC in Type S.

The molecular weight of free CMC was examined to further understand the adsorption mechanism of CMC. GPC chromatograms of the CMC released from 3D-SLISE slurries (Type S and Type E) and the Pullulan calibration curve are shown in **Figure S4**. The molecular weight distribution of CMCs was evaluated by extrapolation, including those above 1,600,000, since a large percentage of CMCs were outside the range of the calibration curve. In both Type S (Figure S4b) and Type E (Figure S4c), the molecular weight of free CMC shifted toward the high molecular weight side after 3 months of refrigerated storage, with the amount of change being smaller for Type E. It was estimated that the high molecular weight molecules that contributed to the dispersion were released. The free CMC in Type S had a lower molecular weight than the raw material, and a low molecular weight distribution (around 10,000 to

100,000) was observed (Figure S4d). It was assumed that the high molecular weight contributed to the dispersion.

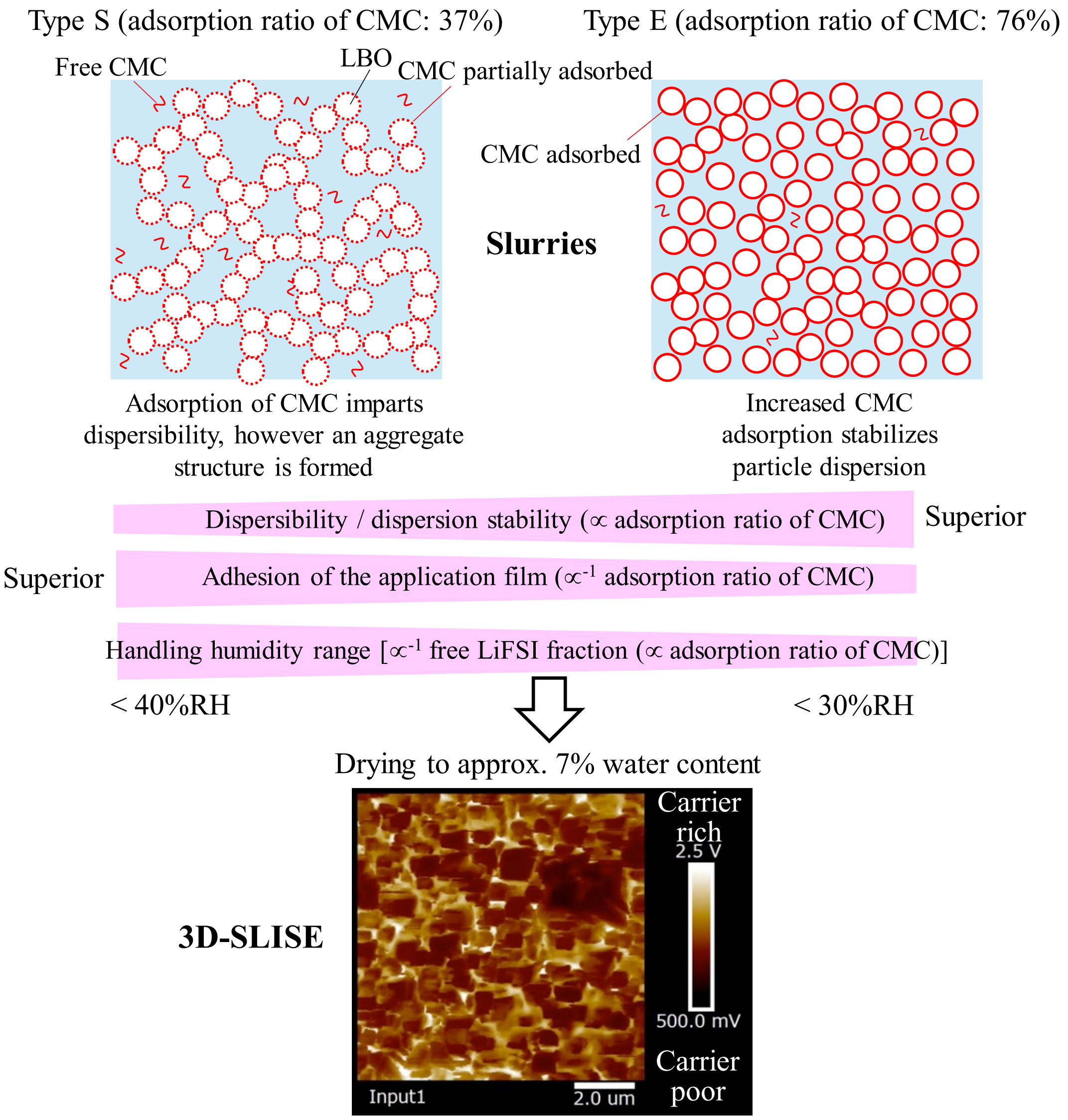

Figure S5. Estimated dispersion mechanism of 3D-SLISE slurry and 3D-SLISE formed by removing excess water [scanning microwave impedance microscopy image (capacitive component) of the 3D-SLISE surface (under nitrogen)].

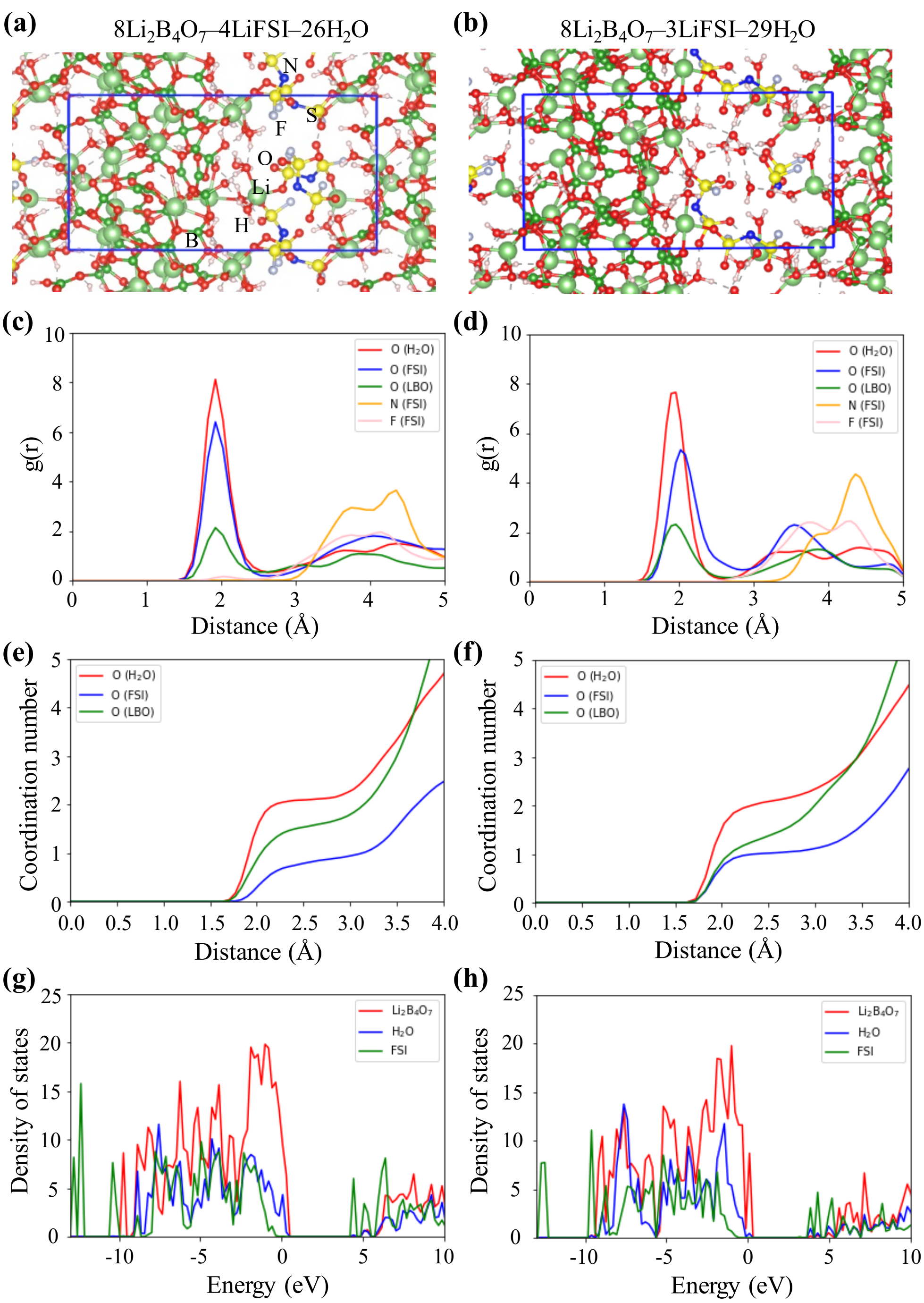

Figure S6. Results of density functional theory (DFT)-based molecular dynamics (DFT–MD) simulations of the interface structure between a-$Li_2B_4O_7$ and LiFSI/H_2O; Supercell models of the a-$Li_2B_4O_7$ and LiFSI/H_2O system treated in first-principles calculations, a) $8Li_2B_4O_7$–4LiFSI–$26H_2O$ and b) $8Li_2B_4O_7$–3LiFSI–$29H_2O$. Radial distribution functions from the counter cation (Li^+) of FSI anions to the O atoms of H_2O, FSI and a-$Li_2B_4O_7$ in the c) $8Li_2B_4O_7$–4LiFSI–$26H_2O$ and d) $8Li_2B_4O_7$–3LiFSI–$29H_2O$ supercell models. Coordination numbers of the O atoms (of H_2O, FSI, a-$Li_2B_4O_7$) to Li atoms in the e) $8Li_2B_4O_7$–4LiFSI–$26H_2O$ and f)

$8Li_2B_4O_7$–3LiFSI–$29H_2O$ supercell models. Partial density of states (PDOS) of H_2O, FSI, and a-$Li_2B_4O_7$ in the g) $8Li_2B_4O_7$–4LiFSI–$26H_2O$ and h) $8Li_2B_4O_7$–3LiFSI–$29H_2O$ supercell models. The PDOS are calculated as the average of 10 structures extracted from 2-ps molecular dynamics trajectories. Both supercells exhibit similar PDOS characteristics.

Density functional theory molecular dynamics (DFT-MD) simulations at the interface between a-$Li_2B_4O_7$ and LiFSI/H_2O structures were performed to understand the good dispersion in water and electrochemical effects of structure formation (**Figure S6**). Two systems with different water/salt ratios, $8Li_2B_4O_7$–4LiFSI–$26H_2O$ (Figure S6a) and $8Li_2B_4O_7$–3LiFSI–$29H_2O$ (Figure S6b), were used to model a-$Li_2B_4O_7$ and LiFSI/H_2O interface structures. The a-$Li_2B_4O_7$ surface containing B atoms with dangling bonds reacted rapidly with H_2O molecules. The radial distribution function (RDF) around the Li atom (counter cation of FSI) and the corresponding coordination number in the LiFSI/H_2O layer are shown in Figure S6c-S6f. The calculated RDF (Figure S6c and S6d) indicate that the first peak of Li^+–O is located at 1.92 (1.96), 1.92 (2.03) and 1.92 (1.96) Å for the O atoms of H_2O, FSI and $L_2B_4O_7$ in $8Li_2B_4O_7$–4LiFSI–$26H_2O$ ($8Li_2B_4O_7$–3LiFSI–$29H_2O$), respectively. The coordination numbers show that all Li atoms in the LiFSI/H_2O-layer are surrounded by about four oxygen atoms. Li atoms in the LiFSI/H_2O layer, particularly those near the interface with a-$Li_2B_4O_7$, bond not only to the oxygen atoms in FSI and H_2O molecules but also directly to the oxygen atoms on the a-$Li_2B_4O_7$ surface. Other Li atoms in the LiFSI/H_2O layer are coordinated by four oxygen atoms of FSI and H_2O.

The interface model for a-$Li_2B_4O_7$ / (H_2O + FSI) differs from the experimental molar ratio from that employed in the ab initio calculation. However, the reactivity of the a-$Li_2B_4O_7$ surface with water does not appear to be strongly dependent on the water molar ratio. Furthermore, even in model calculations in water-rich systems, the potential window is found to be

determined by FSI anions rather than water molecules. This effect is likely to be even more pronounced at the 3D-SLISE interface, where the water to LiFSI molar ratio is much lower.

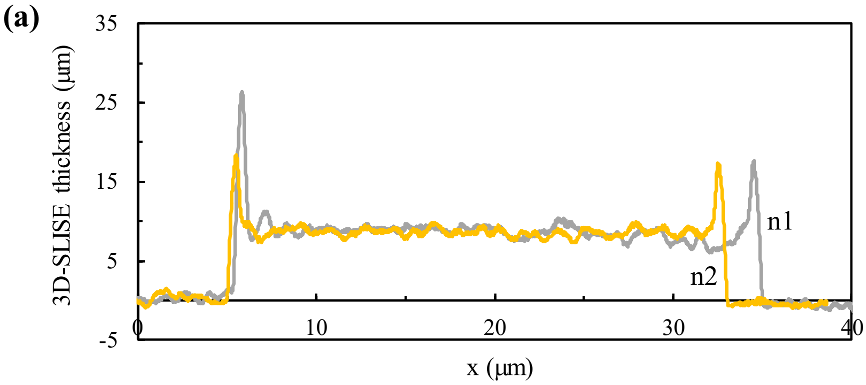

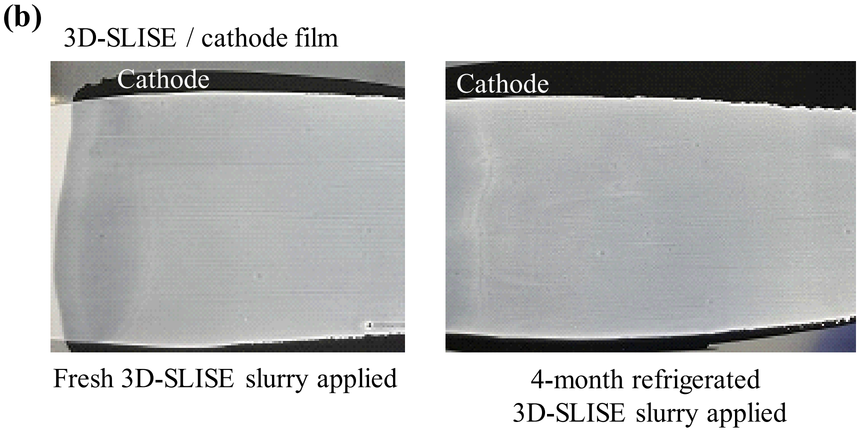

Figure S7. a) Thickness profiles of thin 3D-SLISE films (Type S) applied on Al foil (perpendicular to the direction of application, measured by a laser displacement meter after drying). b) Multilayer films fabricated by the application of fresh and 4-month refrigerated (4°C) 3D-SLISE slurries on cathodes.

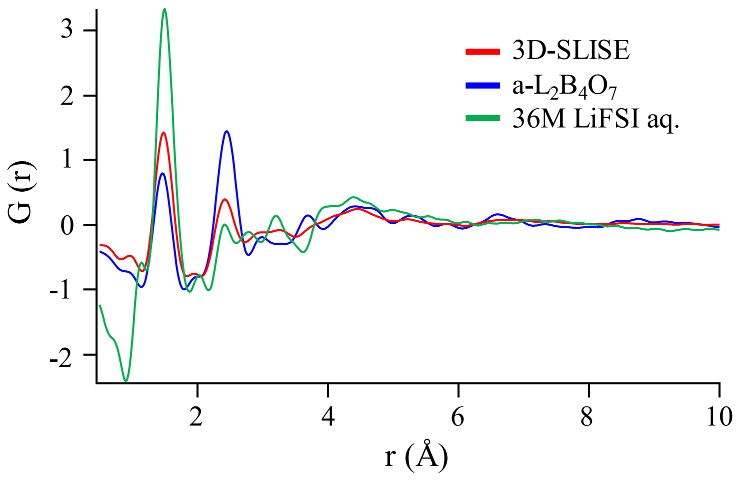

Figure S8. Reduced two-body distribution functions of 3D-SLISE, a-$Li_2B_4O_7$, and an aqueous solution of LiFSI (36 M) constructed using X-ray total-scattering data.

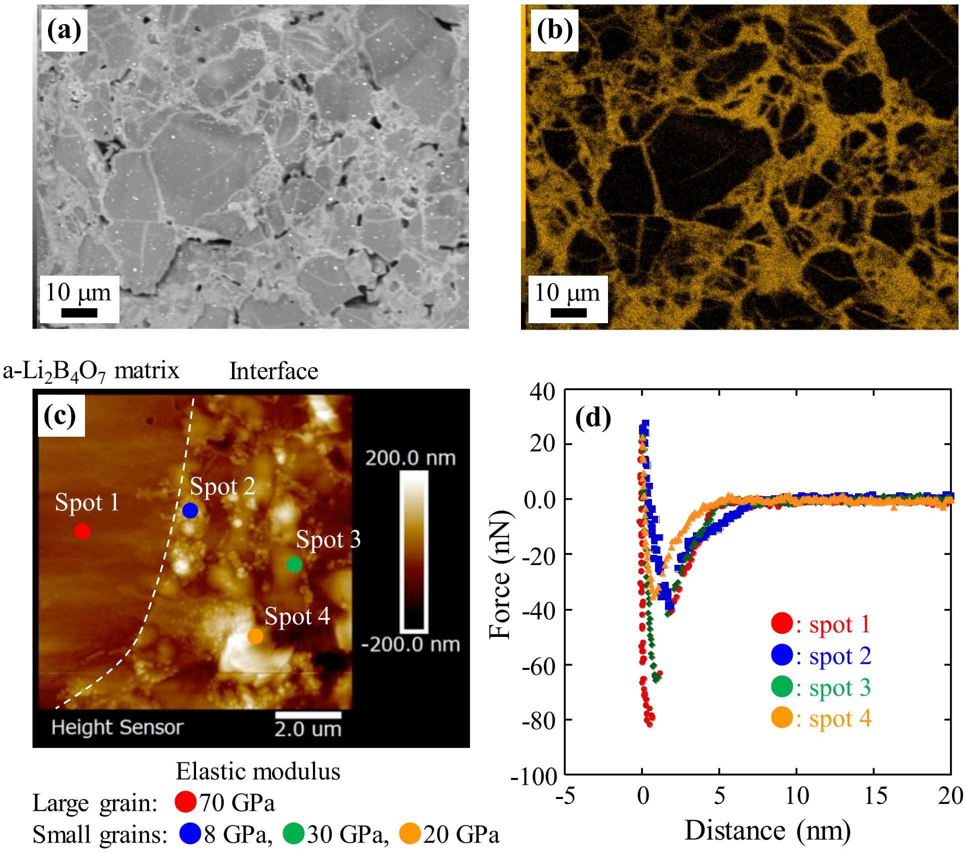

Figure S9. a) SEM image, b) FSI⁻-derived S K-edge intensity image (obtained by EDS), c) AFM image showing the elastic modulus at different spots, and d) the force curve at each spot of the AFM image (elastic modulus is calculated by the Johnson–Kendall–Roberts model) for polished cross-sections of an 3D-SLISE pellet. In order to clearly separate the matrix and conduction layer when obtaining the force curve, a sample with a coarse matrix was polished and used for measurement.

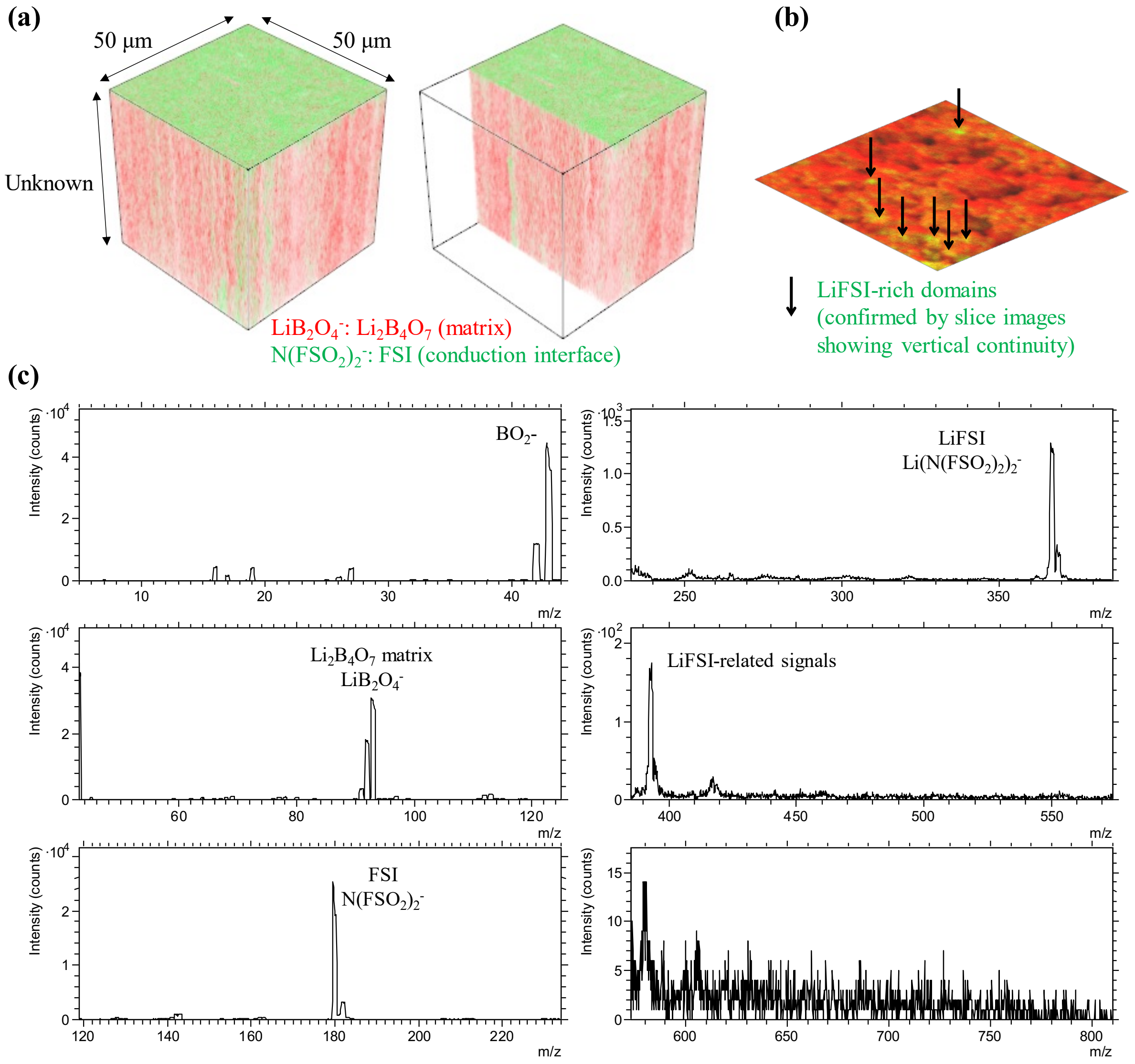

Figure S10. a) 3D composition imaging of 3D-SLISE pellet acquired by Ar-GCIB/TOF-SIMS, b) 2D composition imaging of a certain slice plane, c) mass spectra of the domains that gave strong FSI-derived signals.

Figure S10 shows a 3D composition distribution image acquired while etching a 3D-SLISE pellet from the surface. The top surface is FSI-rich, which is thought to be derived from LiFSI dissolved in surface moisture during prior atmospheric handling (Figure S10a). Upon further etching, compositional regions of mixed $Li_2B_4O_7$ matrix and LiFSI appeared. The domains where the LiFSI signal was particularly strong and vertically linked were considered to be the conduction interface of 3D-SLISE (Figure S10b), and these signals were averaged and

reconstructed as mass spectra (Figure S10c). Although it cannot be concluded based on these results alone, boron may be present at the conduction interface of 3D-SLISE due to the reactions described above (Table S2-1).

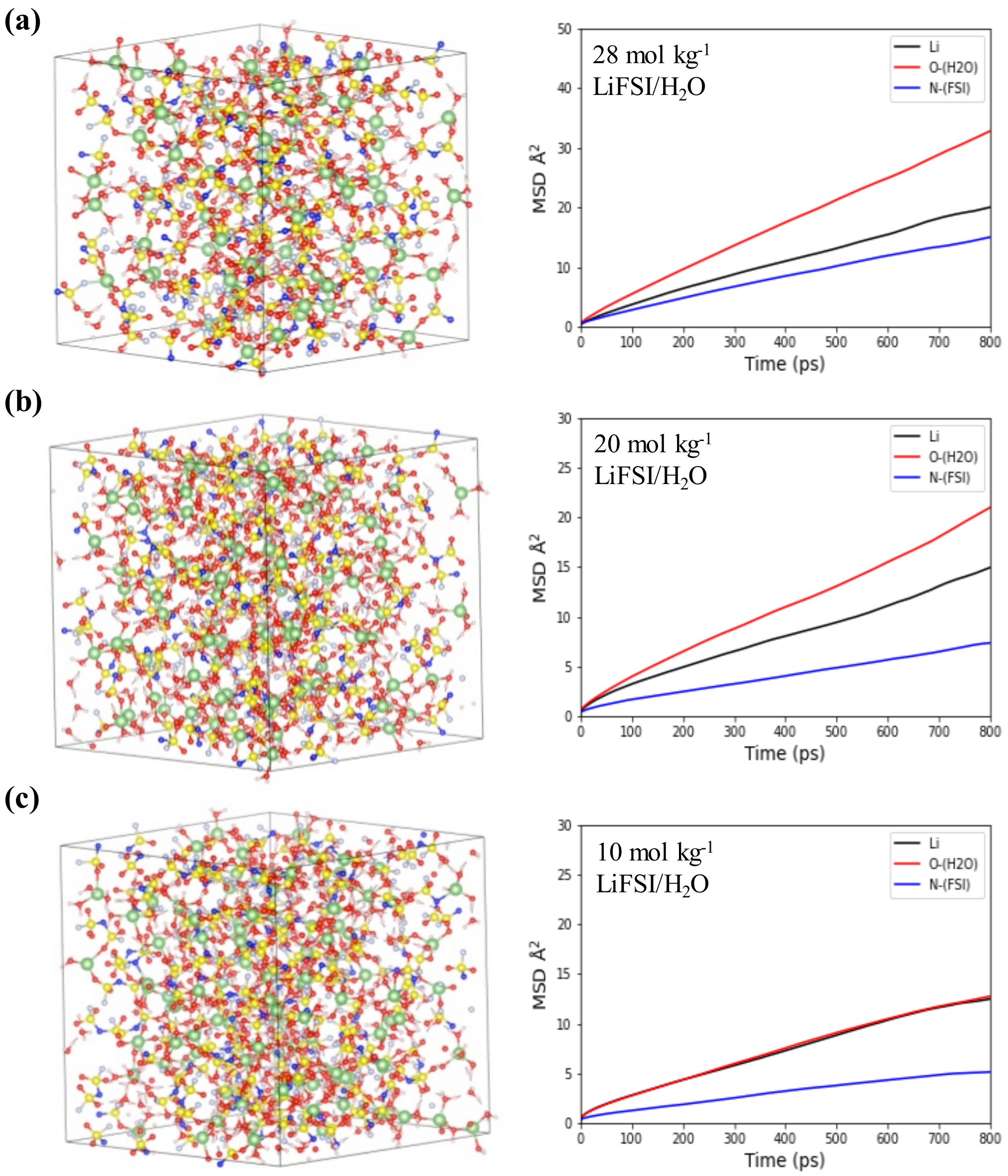

Figure S11. (left) Simulation cells of the conduction layer model (LiFSI/H_2O eutectic system) used in the machine learning potential molecular dynamics (MLP-MD) calculation, (right) mean square displacements (MSD) of Li, H_2O and FSI molecules at 300 K obtained from the calculation, a) 28 mol kg^{-1}, b) 20 mol kg^{-1} and c) 10 mol kg^{-1}. The simulation cells contain 80LiFSI-160H_2O, 108LiFSI-300H_2O and 54LiFSI-300H_2O, respectively.

To investigate the conduction mechanism of Li-ions at the 3D-SLISE interface, machine learning potential molecular dynamics (MLP-MD) calculations were performed for the

LiFSI/H_2O system (10–28 mol kg^{-1}) (**Figure S11, Table S3**). Here, we modeled a LiFSI/H_2O rich layer in the 3D-SLISE with only H_2O and LiFSI molecules by MLP-MD simulations.

Table S3. Calculated self-diffusion coefficients of Li, H_2O, and FSI molecules for 28, 20, and 10 mol kg^{-1} LiFSI/H_2O eutectic model systems at 300 K using the MLP-MD method. The unit of self-diffusion coefficient is 1.0×10^{-11} m^2 s^{-1}.

LiFSI concentration (mol kg^{-1})	28	20	10
Li	2.3	2.9	3.9
H_2O	2.4	4.1	6.7
FSI	0.93	1.4	2.6

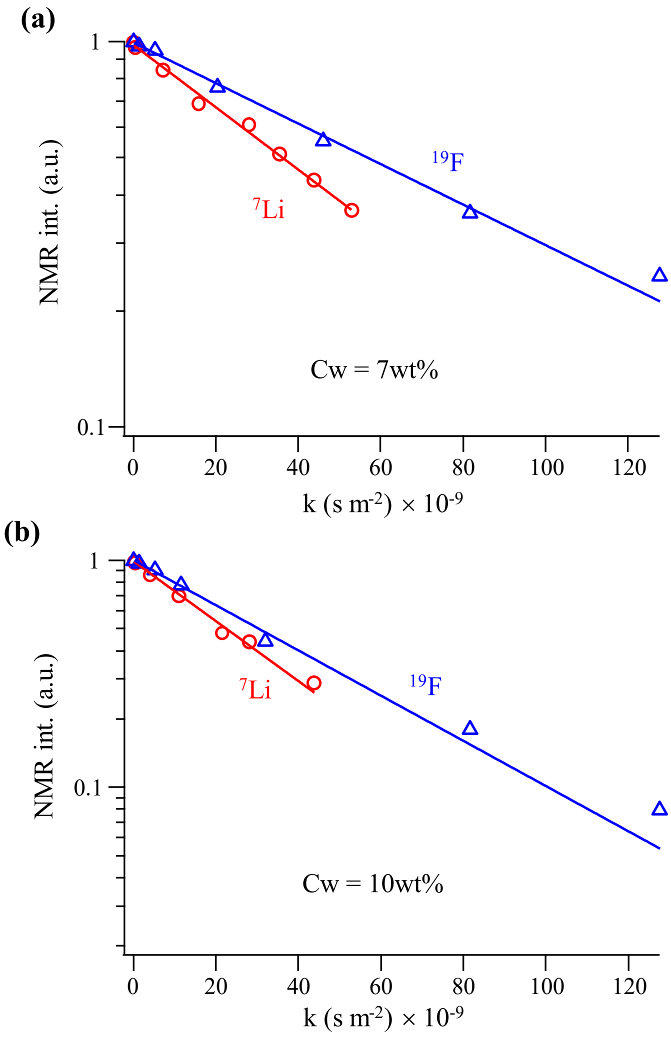

Figure S12. ^{7}Li-NMR and ^{19}F-NMR diffusion plots obtained for 3D-SLISE.

(a) Cw = 7wt%, (b) Cw = 10wt%

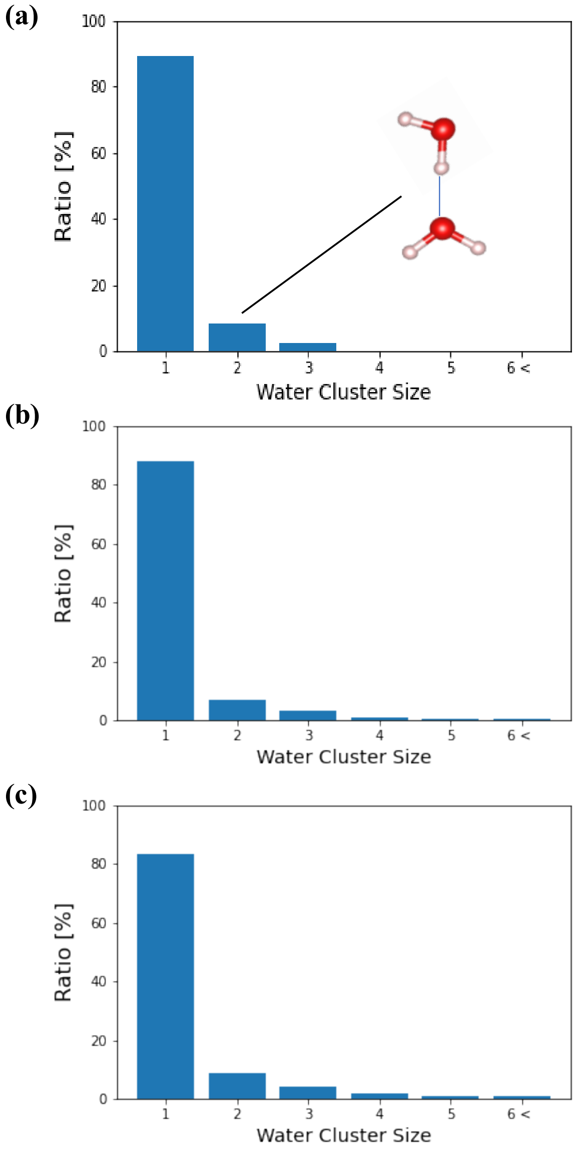

Figure S13. Histograms of H_2O clusters in the LiFSI/H_2O systems, a) 28 mol kg^{-1}, b) 20 mol kg^{-1} and c) 10 mol kg^{-1}, respectively; H_2O clusters were defined by whether each water molecule is hydrogen bonded to other water molecules. Hydrogen bonds are typically identified by the following geometric criteria: the distance between donor-acceptor must be less than a specified cutoff value (usually 3.5 Å), and the angle between donor hydrogen and acceptor must be in the range of 150° to 180°.

Cluster analysis of hydrogen bonds between water molecules in this composition range (**Figure S13**) reveals that 80~90% of the water molecules exist as isolated water molecules

without hydrogen bonds with other water molecules. The structure of these isolated water molecules is thought to have a significant effect on the properties of the electrolyte, such as the expansion of the potential window. We believe that this kind of isolated water structure is also realized in the 3D-SLISE.

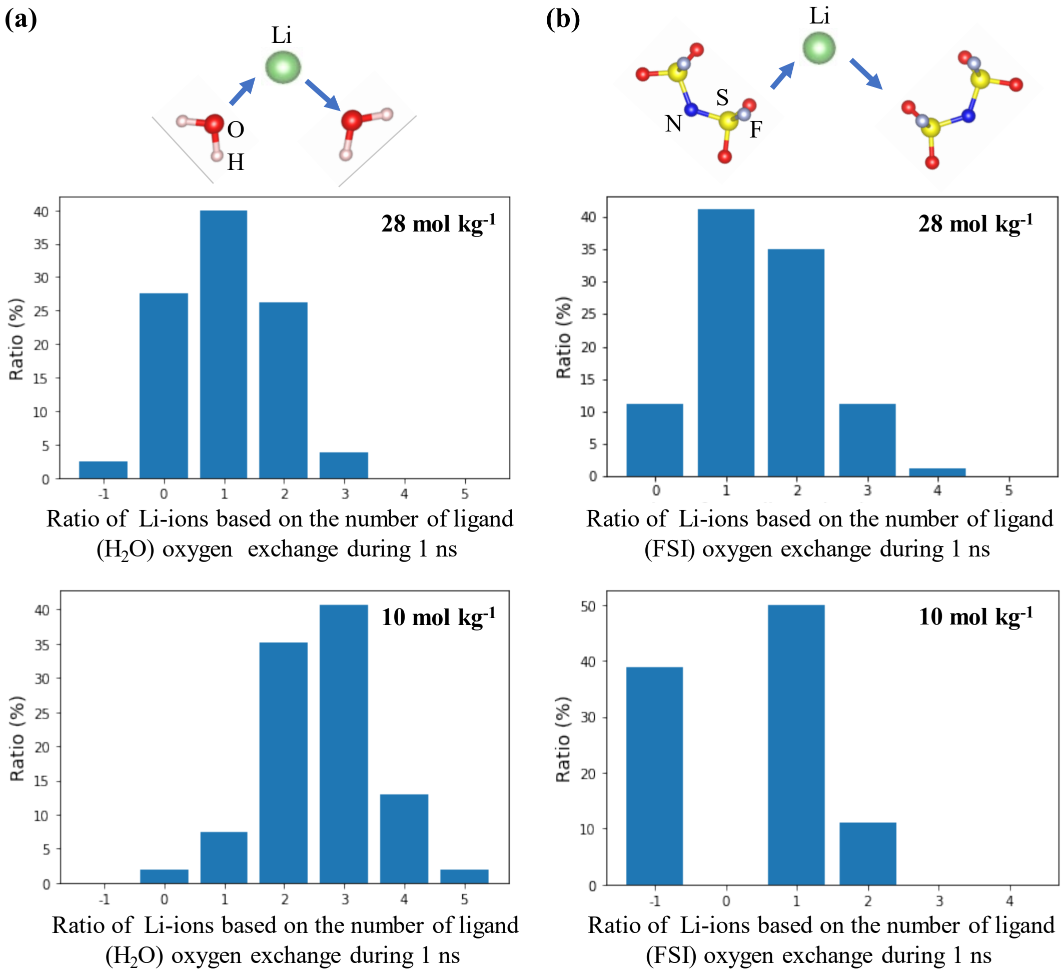

Figure S14. Histograms showing the ratio of Li-ions based on the exchange frequency of coordinated (a) H_2O and (b) FSI oxygen atoms during 1 ns MLP-MD calculations at 300 K. '-1' represents the ratio of Li-ions in the system that were never coordinated by oxygen of H_2O (anion) molecules. For example, the H_2O exchange histogram for 28 mol kg^{-1} LiFSI-H_2O (Figure S14a) shows that approximately 40% of Li-ions exchange 1-coordinated H_2O within 1 ns.

MLP-MD calculations were used to analyze the dynamic behavior of Li-ion diffusion in the LiFSI/H_2O eutectic system as a 3D-SLISE conduction interface model. **Figure S14** shows histograms of the ratio of Li-ions based on the exchange frequency of coordinated H_2O and FSI anion oxygen exchanges during the 1 ns simulation in the 28 (10) mol kg^{-1} LiFSI-H_2O system.

During 1 ns, the Li-ion exchanged the coordinating water molecule an average of 1.03 (2.61) times.

When bound to the FSI anion, it exchanged coordination molecules an average of 1.51 (0.72) times during 1 ns. This indicates that Li-ions exchange all coordination molecules within 1.57 (1.20) ns, suggesting that the diffusion mechanism of Li-ions is closer to hopping-type conduction than vehicle-type conduction. However, it should be noted that the dynamics of H_2O strongly influences the diffusion of Li-ions, as have been observed that the tendency for Li-ion diffusion coefficient to increase in systems with high water content in MLP-MD results (Table S3) and pulsed field gradient NMR measurements (Table 3, Figure S12).

On the other hand, the calculations show that the diffusion of Li-ions is greater in systems with a higher proportion of H_2O molecules, and the ligand exchange of Li-ions occurred more frequently. This may be due to the fact that H_2O molecules with high kinetic energy transfer their energy to Li-ions during ligand exchange, enhancing the mobility of Li-ions. Therefore, water mobility strongly affects Li-ion conduction in LiFSI/H_2O eutectic systems, and the conduction mechanism cannot be explained only by a pure hopping type.[8]

At the conduction interface of 3D-SLISE, CMC, boron, and $Li_2B_4O_7$ are present, and Li-ions are expected to diffuse in the 3D interfacial network through the above conduction mechanism. On the other hand, water molecules anchored by Li at the $Li_2B_4O_7$ interface prevent H_2O molecules from acquiring the kinetic energy necessary for Li-ion diffusion. This is the reason why the diffusion coefficient of the 3D-SLISE system is lower than that of the pure liquid system.

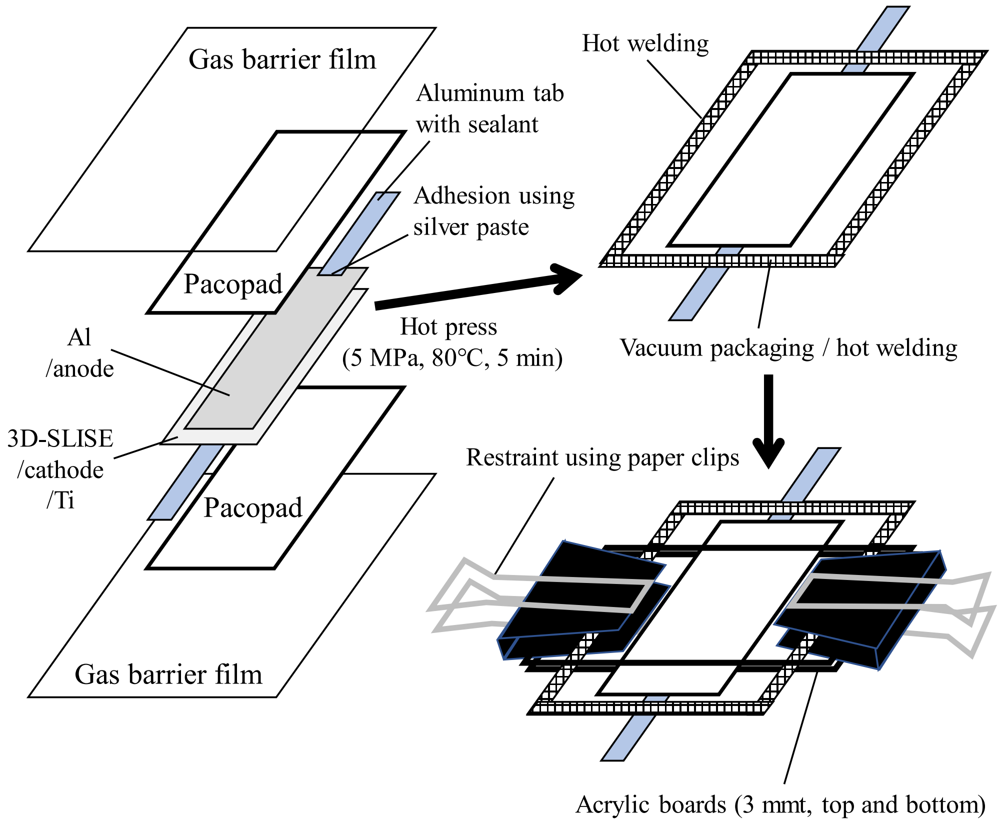

Figure S15. The schematic of the procedure for making a skeleton battery in air (25 °C, 30% RH).

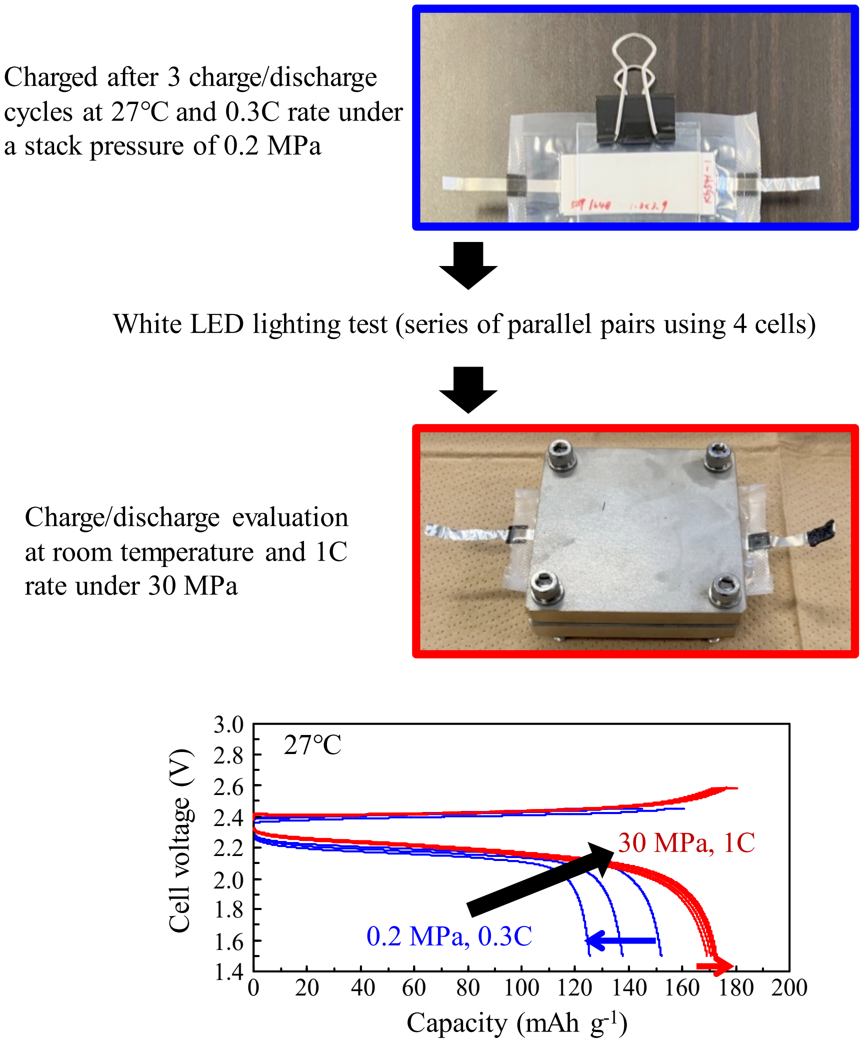

Figure S16. Characteristics of skeleton batteries made in air. The capacity gradually decreases during operation at 0.3C at room temperature due to insufficient contact at each interface under a restraint pressure of 0.2 MPa. After the white LED lighting test for 4 hours, if the stack pressure is increased to 30 MPa, the characteristics become more stable.

Experimental Section

Agglomeration Diameter Analysis: Ultra-small angle X-ray scattering (USAXS) measurements were performed using a Nanopix small-angle X-ray scattering system manufactured by Rigaku. Cu Kα (1.54 Å, 40 kV / 30 mA) was used as the light source, and the camera length was set to about 1800 mm. A 2PHF (low resolution/high intensity) optical system was used. For shearing, the gap and shear rate were set to 150 μm and 500 s^{-1}, respectively.

Dynamic Viscoelastic Analysis: The shear rate dependence of viscoelasticity was analyzed using a dynamic viscoelasticity measurement system (MCR702, Anton Paar Co.) with steady flow: 0.01-1000 s-1, oscillating flow gap: 0.8 mm, and temperature: 25°C. Shear rate was applied up to 1000 s-1 and measured by decreasing the velocity.

Composition analysis of 3D-SLISE interface: Analysis focused on the conduction interface of 3D-SLISE was performed by time-of-flight secondary ion mass spectrometry (TOF-SIMS) using TOF-SIMS 5 (ION-TOF Co.) under the following conditions. Primary ion: Bi^{3+} (25 kV, 0.06 pA), mode: fast imaging mode (high spatial resolution mode), area: 50 μm × 50 μm , 256 × 256 pixels, 3 frames/cycle, argon gas cluster ion beam (Ar-GCIB): Ar_{1200}^{+}, 15 kV, 500 μm × 500 μm, 29. 33 sec (20 frames)/sputter cycle.

Morphological Analysis: For the detailed cross-sectional morphological analysis of 3D-SLISE, 1-mm-thick pellets of 3D-SLISE-coated films, fabricated by the compaction (under 20 MPa of pressure) of 3D-SLISE powder peeled off from the base material (Al) after the natural drying of 3D-SLISE films, were used. These pellets were placed between 0.5-mm-thick plastic plates for reinforcement, followed by razor and ultramicrotome cutting. Finally, the cross-sections were polished using a JEOL IB-19520CCP cross-section polisher at 8.0 kV for 3 h (CP processing). After CP processing, the cross-sections were immediately treated by a Neoc-ST

osmium coater (MEIWAFOSIS, Inc.) for conductivity measurements. A JEOL JXA-8530F instrument at an acceleration voltage of 20 kV and irradiation current of 2.4×10^{-9} A was used for SEM. Auxiliary compositional imaging was conducted using auxiliary energy-dispersive X-ray spectroscopy with a field-emission electron probe micro analyzer (wavelength-dispersive X-ray spectroscopy). AFM measurements of the CP-processed cross-sections followed the conditions described in the main text.

[1] L. Suo, O. Borodin, T. Gao, M. Olguin, J. Ho, X. Fan, C. Luo, C. Wang, K. Xu, *Science* **2015**, *350*, 938.

[2] Y. Yamada, K. Usui, K. Sodeyama, S. Ko, Y. Tateyama, A. Yamada, *Nat. Energy* **2016**, *1*, 1.

[3] X. Hou, T.P. Pollard, X. He, L. Du, X. Ju, W. Zhao, M. Li, J. Wang, E. Paillard, H. Lin, J. Sun, K. Xu, O. Borodin, M. Winter, J. Li, *Adv. Energy Mater.* **2022**, *12*, 2200401.

[4] J.D. Hemry, M.C. Weinberg, D.R. Uhlmann, *J. Mater. Sci.* **1998**, *33*, 3853.

[5] W.C. Lepry, S.N. Nazhat, *Adv. NanoBiomed Res.* **2021**, *1*, 2000055.

[6] a) E.Z. Casassa, A.M. Sarquis, C.H. Van Dyke, *J. Chem. Edu.* **1986**, *63*, 57; b) B. Rohrig, *Chem. Matters.* **2004**, *22*, 13; c) K. Parida, V. Kumar, W. Jiangxin, V. Bhavanasi, R. Bendi, P.S. Lee, *Adv. Mater.* **2017**, *29*, 1702181.

[7] a) M. Huang, Y. Hou, Y. Li, D. Wang, L. Zhang, *Designed monomers and polymers* **2017**, *20*, 505; L. Giuliani, C. Genova, V. Stagno, L. Paoletti, A.L. Matulac, A. Ciccola, M.D. Fazio, S. Capuani, G. Favero, *Gels* **2024**, *10*, 455.

[8] Y. Okuno, *J. Phys. Chem. B* **2025**, *129*, 3639.
